# Electroacupuncture ameliorates cerebrovascular impairment in Alzheimer's disease mice via melatonin signaling

**DOI:** 10.1111/cns.14027

**Published:** 2022-11-15

**Authors:** Yimin Jiang, Yunshi Lin, Yuhang Tan, Xinkai Shen, Meihua Liao, Huan Wang, Nannan Lu, Feng Han, Nenggui Xu, Chunzhi Tang, Juxian Song, Rongrong Tao

**Affiliations:** ^1^ South China Research Center for Acupuncture and Moxibustion Guangzhou University of Chinese Medicine Guangzhou China; ^2^ College of Life Science and Technology Dalian University Dalian China; ^3^ Department of Neurology and Neurological Sciences Stanford University School of Medicine Stanford California USA; ^4^ Key Laboratory of Cardiovascular and Cerebrovascular Medicine, School of Pharmacy Nanjing Medical University Nanjing China

**Keywords:** Alzheimer's disease, cerebrovascular impairment, electroacupuncture at ST36 acupoint, endothelial cell, melatonin signaling

## Abstract

**Aims:**

Cerebrovascular impairment contributes to the pathogenesis of Alzheimer's disease (AD). However, it still lacks effective intervention in clinical practice. Here, we investigated the efficacy of electroacupuncture (EA) in cerebrovascular repair in 3xTg‐AD mice and its mechanism.

**Methods:**

3xTg‐AD mice were employed to evaluate the protective effect of EA at ST36 acupoint (EAST36). Behavioral tests were performed to assess neurological disorders. Laser speckle contrast imaging, immunostaining, and Western blot were applied to determine EAST36‐boosted cerebrovascular repair. The mechanism was explored in 3xTg mice and endothelial cell cultures by melatonin signaling modulation.

**Results:**

EAST36 at 20/100 Hz effectively alleviated the olfactory impairment and anxiety behavior and boosted cerebrovascular repair in AD mice. EAST36 attenuated cerebral microvascular degeneration in AD mice by modulating endothelial cell viability and injury. Consequently, the Aβ deposits and neural damage in AD mice were reversed after EAST36. Mechanistically, we revealed that EAST36 restored melatonin levels in AD mice. Melatonin supplement mimicked the EAST36 effect on cerebrovascular protection in AD mice and endothelial cell cultures. Importantly, blockage of melatonin signaling by antagonist blunted EAST36‐induced cerebrovascular recovery and subsequent neurological improvement.

**Conclusions:**

These findings provided strong evidence to support EAST36 as a potential nonpharmacological therapy against cerebrovascular impairment in AD. Further study is necessary to better understand how EAST36 treatment drives melatonin signaling.

## Graphical abstract

Cerebrovascular protection has been recognized as an alternative target for Alzheimer's disease (AD) prevention and therapy based on the strong contribution of cerebrovascular dysfunction to AD progression. This work addressed that EA at ST36 (EAST36) works as an efficient approach to boost cerebrovascular repair and thereby neurological improvement against AD progression by activating melatonin signaling.

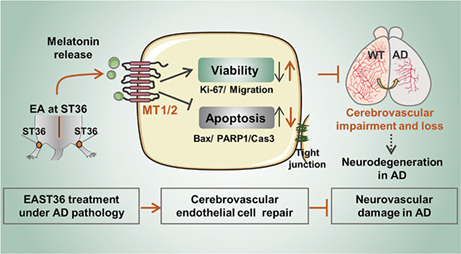

## 1 INTRODUCTION

Alzheimer's disease (AD) is a devastating neurodegenerative disease threatening our aged population globally, which is featured by progressive neurological symptoms including anxiety, depression, olfactory loss, memory loss, and cognitive decline.^1^ Despite intensive efforts from pharmacological research, AD is still considered incurable in clinical practice.^2^, ^3^, ^4^, ^5^ Focusing on risk assessment and intervention of early events has been recognized as key to AD treatment. Recently, emerging findings addressed cerebrovascular dysfunction as one major contributor to AD progression.^6^, ^7^ Blood–brain barrier breakdown and cerebral hypoperfusion occur years before AD onset. The strong correlation between cerebrovascular impairment and cognitive decline in AD was also verified.^8^, ^9^ Thus, cerebrovascular impairment potentially serves as an early indicator of AD, but also provides targets to prevent subsequent neurological deterioration in AD. Delivery of vascular growth factors has been wildly studied in preclinical research to boost angiogenesis and cerebrovascular recovery. However, it remains unavailable in the clinic partially due to the risk of multiple adverse effects.^10^, ^11^

Besides pharmacological intervention, acupuncture has been widely practiced as non‐pharmacological medicine for disease treatment with low cost and high safety.^12^ Combined with electrical current to acupuncture needles, electroacupuncture (EA) provides a more accurate and quantitatively controlled approach for acupuncture treatment.^13^, ^14^ For example, EA at Zusanli (ST36), a commonly applied acupoint on the leg, could drive distinct vagal‐adrenal axis or spinal‐sympathetic axis to boost anti‐inflammation effect in EA intensity‐dependent manner.^15^, ^16^ Impressively, preclinical and clinical studies have elucidated the neuroprotective effect of acupuncture treatment in diseases that are difficult to be managed with conventional pharmacological therapies.^14^, ^17^ Molecularly, acupuncture attenuated AD via modulating autophagy flux in microglia, neuroinflammation, synaptic plasticity, and apoptosis in neural cells.^18^, ^19^ However, the cerebrovascular protection of acupuncture against AD progression remains largely unelucidated.

Melatonin (MT), an endogenously produced hormone, has been increasingly attractive due to its multifunction and great potency in neurovascular protection.^20^ MT attenuates cognitive deficit in AD mice by regulation of mitophagy flux, neuroinflammation, and oxidative stress.^20^, ^21^ Besides neuroprotection, we previously reported MT decreased cerebrovascular endothelial injury and BBB breakdown to establish cerebral homeostasis.^22^, ^23^ However, MT level declined with aging which is more sustained in AD patients.^20^, ^24^ Restoring MT levels suppressed anxiety, insomnia, and cognitive deficit, which also are the major symptoms of AD. Based on these benefits, regulation of MT disturbance by pharmacological and nonpharmacological methods has been anticipated to restore neurovascular function in AD.

Here, to address the benefits of EA treatment on AD pathology, we explored the cerebrovascular protection of EAST36 intervention in 3xTg‐AD mice, and dissected the underlying molecular mechanism via modulating melatonin signaling with agonist MT or antagonist Luzindole treatment both in vitro and in vivo. Our findings strongly suggested EAST36 as one promising approach to boost cerebrovascular repair for AD prevention and treatment.

## 2 MATERIALS AND METHODS

### 2.1 Animals

All animal experiments were approved and performed following the experimental animal care and use guidelines of Guangzhou University of Chinese Medicine (approval No: 20181001). Female 3xTg‐AD mice (Stock No.033930; Jackson Laboratory) at 7, 9, and 13 months old were employed because of the higher severity of AD pathology when compared with age‐matched male 3xTg‐AD mice.^8^, ^25^ Age‐matched female C57BL/6J mice served as the wild‐type control (Experimental Animal Center of Guangzhou University of Chinese Medicine, Guangzhou, China). All animals were housed and bred in stable conditions with 12 h light/dark cycle and free access to water and food. All efforts were made to minimize the suffering of mice.

### 2.2 EAST36 treatment

Mice at 7 months old were allocated to the Sham group and EA group randomly. Mice in the EA group were anesthetized with 3% isoflurane and then sustained narcosis by 1.5% isoflurane, and loosely immobilized by sticky tape and treated with EA stimulation (Huatuo SDZ, China) at bilateral ST36 acupoints for 8 weeks. ST36 acupoints locate at 5 mm below the head of the fibula under the knee joint and 2 mm lateral to the anterior tubercle of the tibia.^15^, ^26^ Acupuncture needles were inserted into bilateral acupoints with a depth of 3 mm and connected to the EA stimulation instrument. EA with dilatational wave stimulation was applied at the high frequency of 20 /100 Hz or low frequency of 2 /10 Hz, an intensity of 1 mA, and 20 min each time. Mice in the Sham group were anesthetized and loosely immobilized by sticky tape without EA. EAST36 treatment was performed 5 times each week for 8 weeks. At the end of treatment, experimental mice at 9 months were used for subsequent analysis.

### 2.3 Drug administration

Mice at 7 months old were randomly divided into vehicle group and MT or Luz group. Mice were orally administrated with MT (M5250; Sigma Aldrich) at a daily dose of 20 mg/kg (5 days/week, 8 weeks) or solvent (1% DMSO in ddH_2_O) as the vehicle treatment. MT receptors antagonist Luzindole (L0316; TCI America) was given at the dose of 10 mg/kg 30 min before EAST36 treatment each time (5 days/week, 8 weeks), and mice in vehicle group were administrated with solvent alone before EAST36 treatment.

### 2.4 Behavioral tests

Food‐finding test,^27^ elevated‐plus maze (EPM),^28^ and Morris‐water maze test^29^ were applied to assess the olfactory symptoms, anxiety‐like symptoms, and cognitive deficit in mice, respectively. The detailed performance was described in the Appendix S1.

### 2.5 Cerebral blood perfusion analysis

Laser speckle contrast imaging (LSCI) was applied to measure the cerebral blood flow with PeriCam PSI System (Perimed Inc) following the manufacturer's instructions. The details were described in the Appendix S1.

### 2.6 MT analysis by ELISA

The mouse brain tissues from the hippocampus and prefrontal cortex were dissected and stored in dry ice immediately at 9:00 am‐11:00 am, which was followed by ELISA detection according to the provided manual of the MT ELISA kit (E4630; BioVision). The details were described in the Appendix S1.

### 2.7 Cell culture and treatment

Mouse brain microvascular endothelial cells (bEnd.3; ATCC) were cultured as previous,^30^ which were followed by Western blot analysis, immunostaining, and migration test.

### 2.8 Migration test

The migration activity of bEnd.3 endothelial cells from each experimental group was evaluated by wounding analysis.^31^ The details were described in the Appendix S1.

### 2.9 Fluorescent immunostaining and Western blot analysis

Fluorescent immunostaining and Western blot analysis were performed as previously described.^22^ The details were described in the Appendix S1.

### 2.10 Statistical analysis

Data collection and analysis were performed by investigators blind to the group assignment. All data are represented as the mean ± SD. Anderson‐Darling, D'Agostino‐Pearson, Shapiro–Wilk, and Kolmogorov–Smirnov normality tests were applied to evaluate data normality. Data from two groups were analyzed with the unpaired, two‐tailed *t*‐test. When data did not pass the variance test, Welch's correction was applied. Data from multiple groups were analyzed with one‐way ANOVA followed by Tukey's test or two‐way ANOVA followed by Sidak test. For data that failed to pass the normality test, the nonparametric Kruskal‐Wallis test with Dunn's test or Mann–Whitney test was used. All statistical analyses were performed with the software Graphpad Prism 8. The difference was considered significant when *p* < 0.05.

## 3 RESULTS

### 3.1 EAST36 alleviated the olfactory impairment and anxiety‐like behavior in 3xTg‐AD mice

AD progression is featured by different stages according to the neurocognitive symptoms.^1^, ^32^ We first performed neurological behavior tests to characterize the symptoms of female 3xTg‐AD mice we used at age of 9 and 13 months (Figure S1). As addressed, moderate olfactory impairment and anxiety‐like behavior were found in 3xTg‐AD mice at age of 9 and 13 months (Figure S1A‐C), which are early clinical symptoms before cognitive loss during AD development.^32^ Results from Morris water maze showed a significant decline in learning and memory in 3xTg‐AD mice at 13 months, rather than the younger ones at 9 months (Figure S1D‐G). This is consistent with our previous observations both in male and female 3xTg‐AD mice at 13 months.^29^, ^33^ These data suggested the 3xTg‐AD mice at 9 months in this study are similar to the early stage of AD according to the definition of AD pathological phase.^32^

**FIGURE 1:**
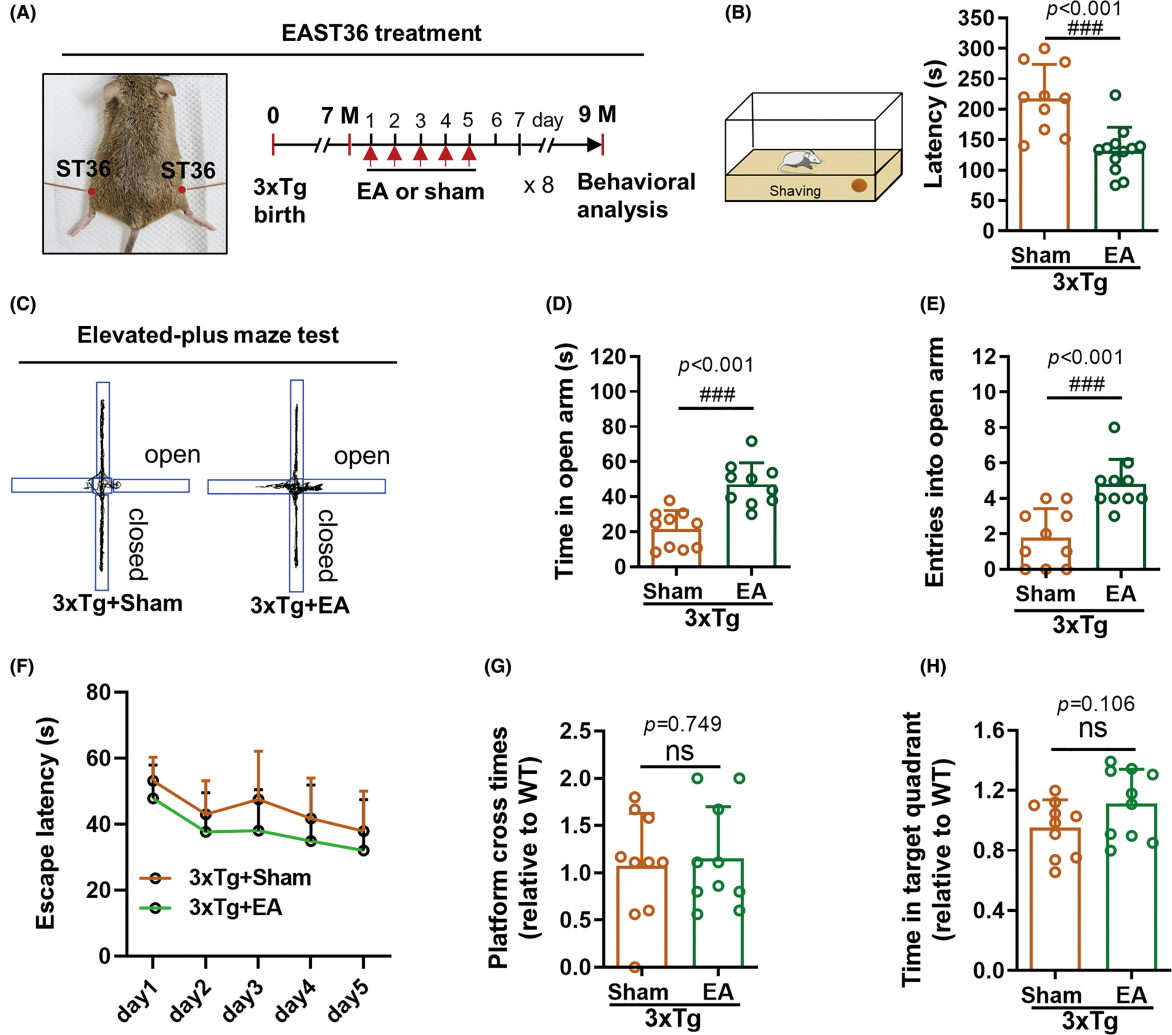
EAST36 alleviated the olfactory impairment and anxiety‐like behavior in 3xTg‐AD mice. (A) Schematic diagram of EAST36 treatment in 3xTg‐AD mice for 8 weeks. (B) Schematic diagram of food finding test for mice and the changes of latency before finding the buried food, which indicates the olfactory‐related neurological impairment in mice. (C‐E) The representative trajectory of mice in elevated‐plus maze test for 5 min, indicating anxiety‐like neurological disorder. It was expressed as time dwelling in the open arm and entries into the open arm. (F‐H) Morris‐water maze test was applied and presented as the learning curve during the training phase (F), times crossing platform location (G), and time in the target quadrant (H) during the probe phase. *n* = 10–12 mice. Data were expressed as Mean ± SD. ^###^ *p* < 0.001 versus sham group. All data passed the normality tests of the Anderson‐Darling, D'Agostino‐Pearson, Shapiro–Wilk, and Kolmogorov–Smirnov tests. Unpaired two‐tailed *t*‐test (B, D, E, G, H) and two‐way ANOVA with Sidak test (F) were used. *n* = 10–12. EA: EAST36; ns: non‐significant.

To explore the effect of EA in AD intervention, EAST36 treatment was conducted in 3xTg‐AD mice for 8 weeks (Figure 1A). As shown by the decrease of food‐finding latency (Figure 1B), EAST36 at the high frequency of 20/100 Hz significantly improved the olfactory ability of 3xTg‐AD mice at 9 months. In the elevated plus maze test, 3xTg‐AD mice showed a preference for closed arms which represent a comfortable and safe environment. EAST36 treatment remarkably promoted exploration in open arms of elevated plus maze compared to the sham group of AD mice (Figure 1C‐E), suggesting the elimination of anxiety‐like disorder in AD mice. In the Morris water maze test, EAST36 showed a mild trend to improve the learning and memory performance of 3xTg‐AD mice, but no significance was found (Figure 1F‐H). Besides, we also explored the EAST36 at low frequency (2 Hz /10 Hz) and found that EAST36 at low frequency was relatively weak in the neurological improvement in AD mice (Figure S2A‐C). Thus, our data demonstrated that EAST36 at high frequency was an efficient intervention to restore the neurological deficits in 3xTg‐AD mice during early stage of AD.

### 3.2 EAST36 attenuated cerebrovascular hypoperfusion and microvascular injury in 3xTg‐AD mice

**FIGURE 2:**
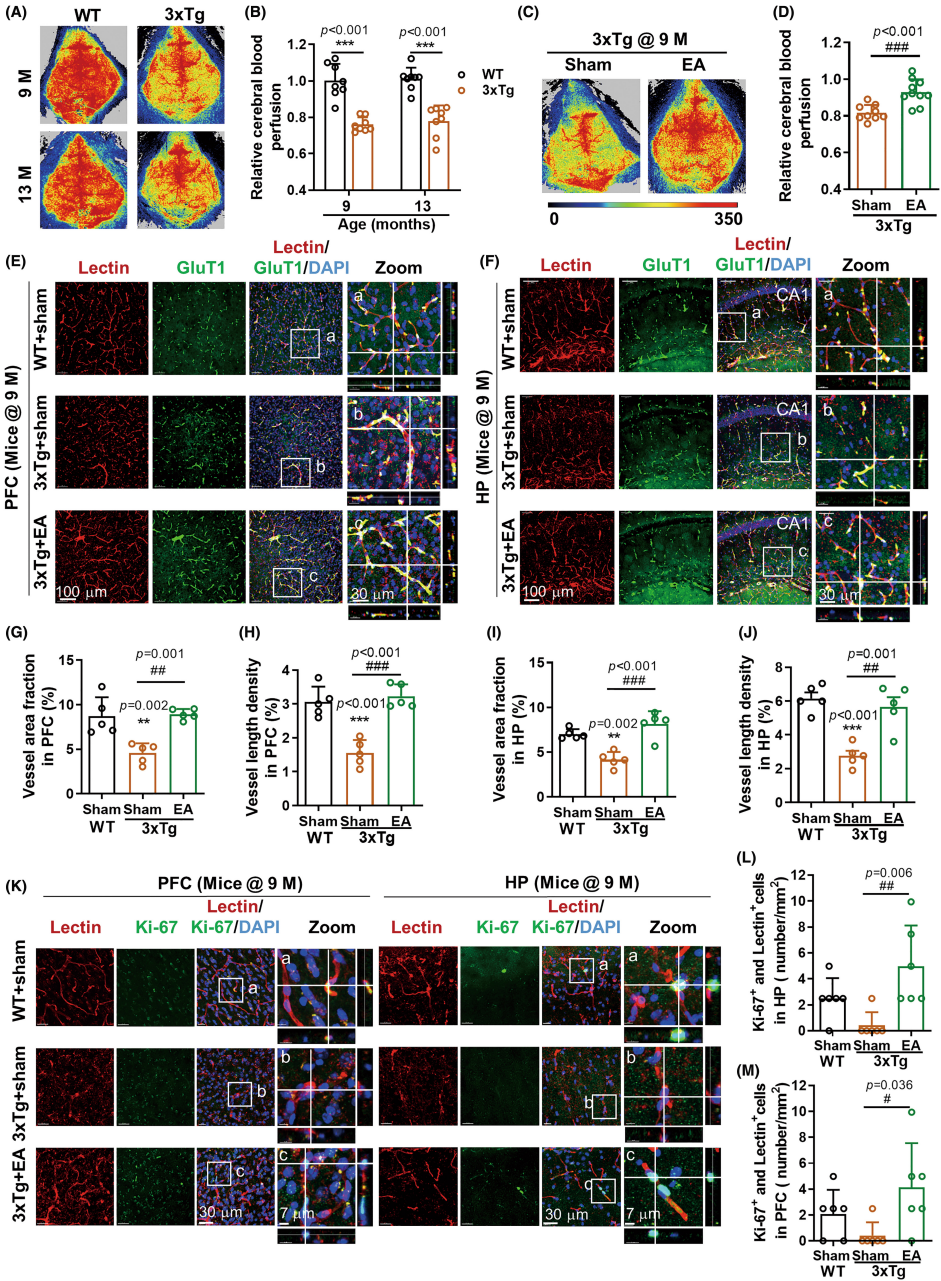
EAST36 attenuated cerebrovascular hypoperfusion and microvascular injury in 3xTg‐AD mice. (A, B) Representative images of cerebral perfusion analysis from mice at 9 and 13 months by laser speckle contrast imaging (LSCI) in vivo, and the quantification normalized to WT group at 9 months (*n* = 8 mice). (C, D) Cerebral perfusion images from 3xTg‐AD mice at age of 9 months and quantitative analysis (*n* = 8 mice). (E, F) Confocal Z‐stack images of cerebral microvessels in the prefrontal cortex (PFC) and hippocampus (HP) regions, which were double indicated with the cerebrovascular endothelial cell markers tomato‐Lectin (Lectin, red) and Glucose transport 1 (GluT1, green). DAPI (blue) was used for nuclei staining. White‐boxed regions were enlarged in the right Zoom panel, respectively. (G‐J) The quantitative analysis of vascular area fraction and vascular length density in the PFC and HP regions. (K) Immunostaining images of Ki‐67 (green) and Lectin (red) in the PFC and HP regions from mice. White‐boxed regions were enlarged in right Zoom panel, respectively. (L, M) The density of proliferative endothelial cells double‐labeled with Ki‐67 and Lectin from PFC and HP regions was measured and plotted (*n* = 5 mice). Data were expressed as Mean ± SD. **p < 0.01, ***p < 0.001 versus WT group, ^#^ *p* < 0.05, ^##^ *p* < 0.01, ^###^ *p* < 0.001 versus 3xTg + sham group. Data in B‐J passed the normality tests of Anderson‐Darling, D'Agostino‐Pearson, Shapiro–Wilk, and Kolmogorov–Smirnov tests. Data in L‐M failed. Two‐way ANOVA with Sidak test (B), unpaired two‐tailed *t*‐test (D), one‐way ANOVA with Tukey's test (G‐J), and Kruskal‐Wallis test with Dunn's test (L‐M) were used. PFC: prefrontal cortex; HP: hippocampus; EA: EAST36.

Cerebrovascular impairment is a crucial pathogenic factor in AD development.^34^ We next explored whether EAST36 promoted cerebrovascular repair in AD treatment. First, we found remarkable and constant cerebral hypoperfusion in 3xTg‐AD mice (Figure 2A‐B), which was consistent with the observation from AD patients.^7^ EAST36 improved cerebral blood perfusion of 3xTg‐AD mice at 9 months (Figure 2C‐D). Consistent with neurological results, no obvious improvement of cerebral perfusion was observed in 3xTg‐AD mice after EAST36 at the low frequency of 2 Hz/10 Hz (Figure S2D).

Then, we dissected the brain to investigate the changes in cerebral microvascular density in 3xTg‐AD mice. Immunostaining of tomato lectin and glucose transporter 1 (GluT1) antibody was used to co‐label the cerebral vessels (Figure 2 E‐F). Tomato lectin binds to the endothelial cell glycocalyx, which has been applied in cerebrovascular imaging.^35^, ^36^ Compared to the age‐matched wild‐type group, a significant alternation of cerebrovascular morphology was found in the 3xTg‐AD group at 9 months, shown as the disintegration and loss of microvascular branches in PFC region and HP region. This cerebrovascular injury in 3xTg‐AD mice was halted by EAST36 (Figure 2 E‐F). Zoomed images presented the representative phenotype of cerebrovessels. These alternations were quantified as the enhancement of vessel area fraction and vessel length density in PFC (Figure 2G‐H) and HP regions (Figure 2I‐J).

We further looked into the changes in cerebrovascular endothelial cell viability, which is dispensable for cerebrovascular angiogenesis and remodeling.^37^ Colocalization of cerebrovascular endothelial marker tomato lectin and proliferative marker Ki‐67 immunostaining was applied to assay the vascular cell proliferation in each group. Compared with wild‐type group, Ki‐67^+^ cell density in the PFC region and HP region of 3xTg‐AD group showed the trend to be suppressed (Figure 2K‐M). EAST36 significantly improved cerebrovascular cell proliferation, as shown by the increased Ki‐67^+^ cell density in lectin‐labeled cerebrovascular endothelial cells in PFC and HP regions. This further suggested the EAST36 protection against cerebrovascular endothelial cell injury in 3xTg mice.

### 3.3 EAST36 suppressed Aβ_1‐42_ deposits and neurovascular damage in 3xTg‐AD mice

**FIGURE 3:**
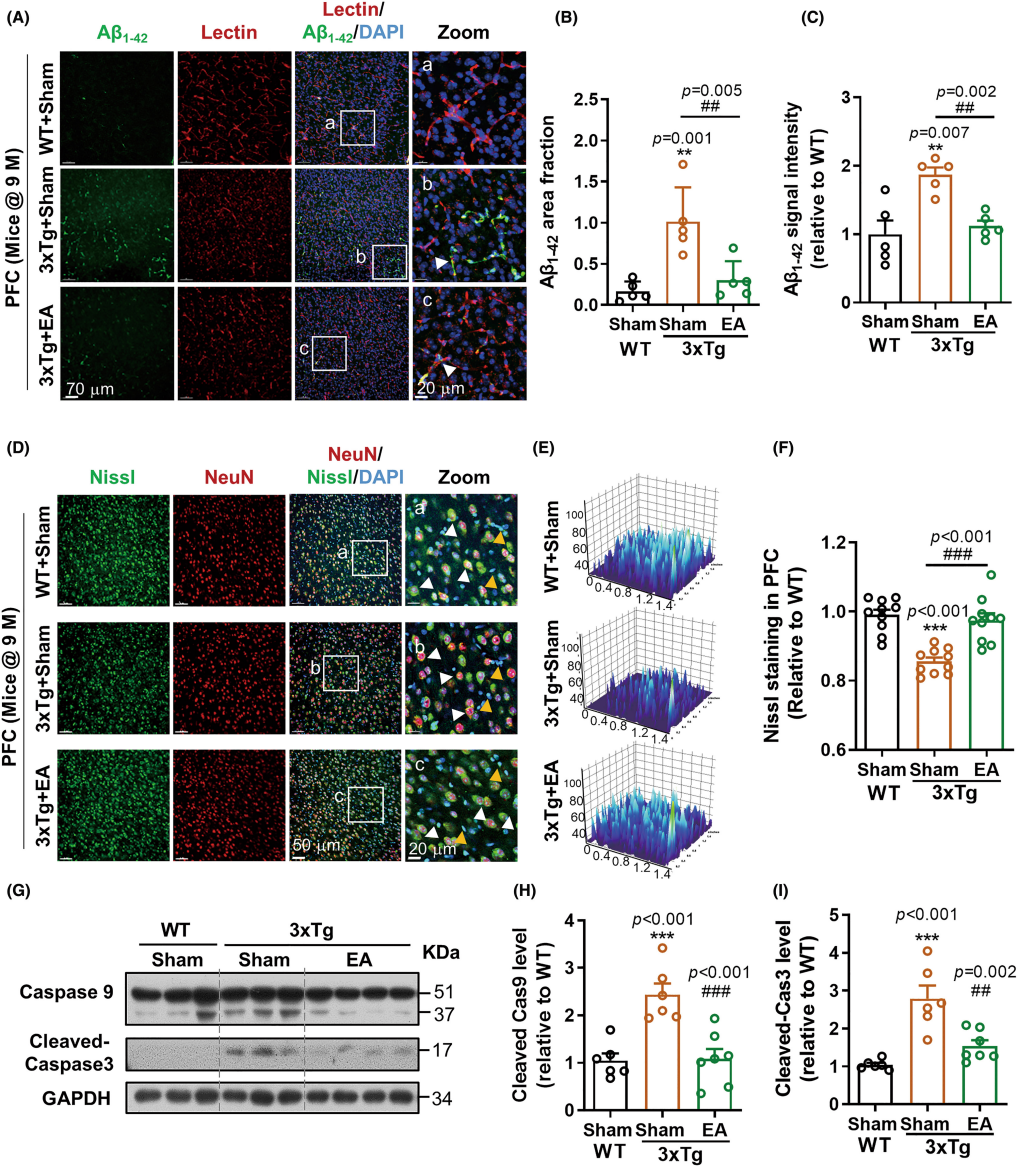
EAST36 suppressed Aβ_1‐42_ deposits and neurovascular damage in 3xTg‐AD mice. (A) Distribution of Aβ deposits in PFC of mice. Aβ deposit was labeled with Aβ_1‐42_ antibody (green). Cerebral microvessels were labeled with Lectin (red). DAPI (blue) was used for nuclei staining. White‐boxed regions were enlarged in the right Zoom panel, respectively. (B, C) Quantitative analysis of Aβ deposit in PFC which was expressed as changes of Aβ deposit area fraction and fluorescence intensity. (D) Representative images of Nissl staining in the PFC region of mice. The co‐staining of Nissl (green), NeuN (red), and DAPI (blue) demonstrated Nissl levels in neuron cells (white arrow). The co‐staining of Nissl (green) and DAPI (blue) without NeuN signal showed Nissl levels in non‐neuron cells (yellow arrow). (E) 3D surface plot analysis of Nissl fluorescence intensity changes in PFC. (F) Nissl level was quantified and plotted as mean fluorescence intensity normalized to WT group (*n* = 10 from 5 mice). (G) Western blotting analysis of Caspase 9 (Pro, 51 kDa; cleaved, 37 kDa) and cleaved‐Caspase 3 in PFC region. GAPDH worked as the sample loading control. (H, I) Protein levels were quantified and normalized to WT group (*n* = 6–8 mice). Data were expressed as Mean ± SD. **p < 0.01, ***p < 0.001 versus WT + Sham group, ^##^ *p* < 0.01, ^###^ *p* < 0.001 versus 3xTg + Sham group. All data passed the normality tests of the Anderson‐Darling, D'Agostino‐Pearson, Shapiro–Wilk, and Kolmogorov–Smirnov tests. One‐way ANOVA with Tukey's test was used. PFC: prefrontal cortex; HP: hippocampus; EA: EAST36.

Aβ deposit is a hallmark and risk of AD progression, which is the result of the imbalance of Aβ production and clearance. Cerebrovascular transportation is an important approach for Aβ clearance from the brain.^38^ To investigate the benefit of EAST36‐promoted cerebrovascular recovery, we next examined Aβ_1‐42_ accumulation in the brain after EAST36 treatment. Immunostaining showed a substantial Aβ_1‐42_ deposit in the PFC (Figure 3A‐C) and HP (Figure S3A‐C) regions from 3xTg‐AD mice. Spatially, Aβ_1‐42_ was majorly distributed at cerebral microvessels of 3xTg‐AD mice at 9 months old (Figure 3A, S3A). This was also supported by distribution profile analysis of Aβ_1‐42_ signal and neuronal marker NeuN signal or microglial marker Iba‐1 signal (Figure S3D‐G). Correspondingly, the microvessel with Aβ deposit was damaged and disintegrated in 3xTg‐AD group. EAST36 suppressed the Aβ_1‐42_ accumulation in the cerebral microvessels, as well as the cerebrovascular disintegration (Figure 3A, S3A). These further indicated the restoration of cerebrovascular function in reducing Aβ deposit after EAST36. Meanwhile, the Aβ precursor protein (APP) expression in the EAST36 group did not change compared to 3xTg‐AD group (Figure S3H‐J).

Subsequently, we explored the neurovascular repair in AD mice, as the consequence of cerebrovascular recovery after EAST36. Fluorescent Nissl staining showed EAST36 suppressed the reduction of Nissl signal in PFC (Figure 3D‐F) and HP (Figure S4A‐C) from 3xTg‐AD group, respectively. Furtherly, the recovery of Nissl level following EAST36 occurred in neuron cells (white arrow), as well as in NeuN‐negative cells (yellow arrow). Western blot analysis showed the activation of another apoptosis indicator caspase9 and caspase3 was also suppressed after EAST36 treatment in the PFC region (Figure 3G‐I) and HP region (Figure S4D‐E) of 3xTg‐AD mice. The enhanced microglial response in 3xTg‐AD group was largely suppressed as presented by the morphological alternation in end points and branch length (Figure. S8). These further demonstrated the prevention of neurovascular injury after EAST36 treatment.

### 3.4 Melatonin supplement mimicked the effect of EAST36 on cerebrovascular repairment in 3xTg‐AD mice

**FIGURE 4:**
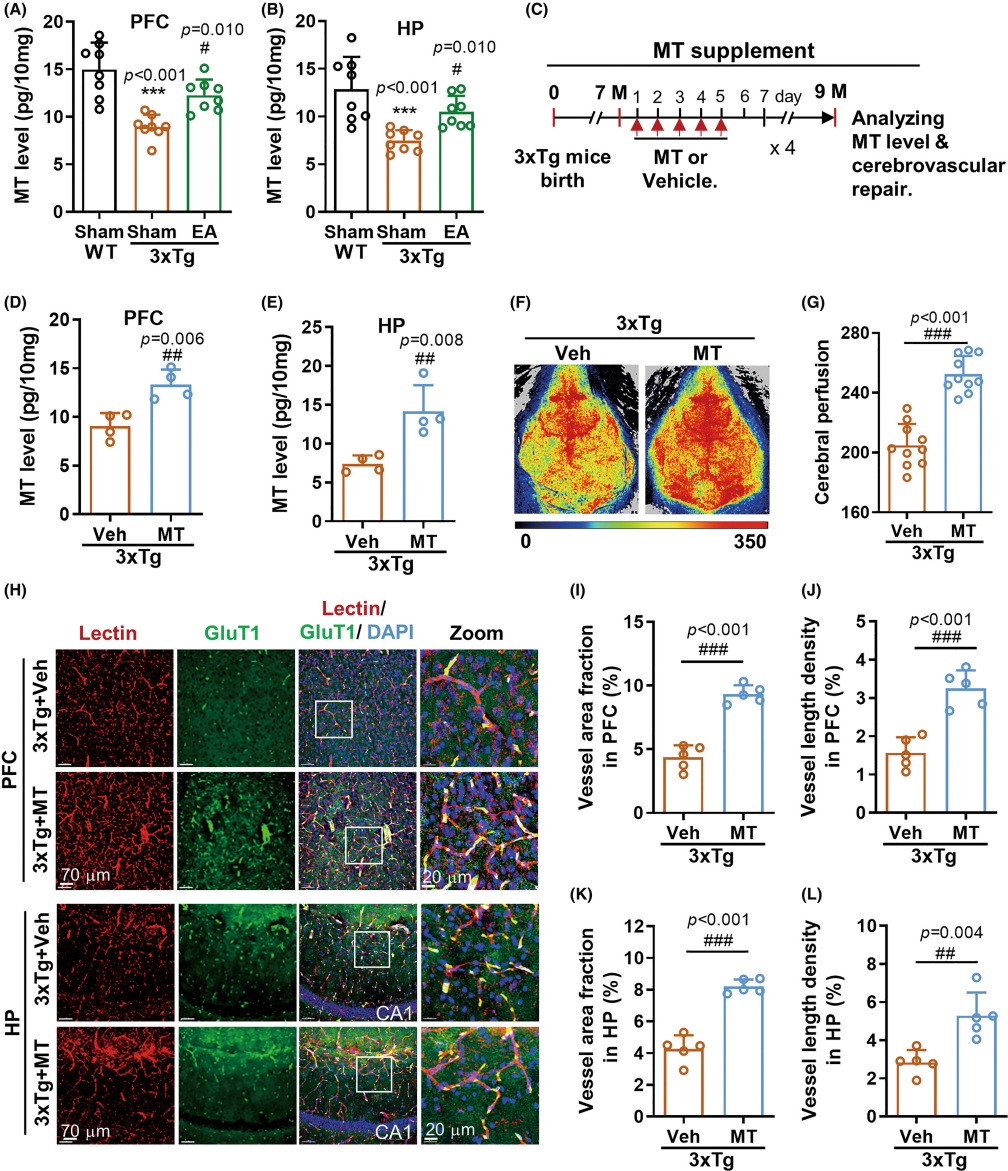
Melatonin supplement mimicked EAST36‐mediated cerebrovascular protection in 3xTg‐AD mice. (A, B) Melatonin level detection in the PFC and HP from each group by ELISA analysis (*n* = 8 mice). ***p < 0.001 versus WT + sham group, ^#^ *p* < 0.05, ^##^ *p* < 0.001 versus 3xTg + sham group. (C) Workflow diagram of MT treatment in mice and following analysis. (D, E) Melatonin level detection in the PFC and HP from each group by ELISA analysis (*n* = 4 mice). (F, G) Cerebral perfusion images by LSCI and quantitative analysis (*n* = 10 mice). (H) Immunostaining images of Lectin (red), GluT1 (green), and DAPI (blue) showed changes in cerebrovascular density in PFC and HP. (I‐L) The quantification of vascular area fraction and length density in the PFC and HP from each group (*n* = 5 mice). Data were expressed as Mean ± SD. ^##^ *p* < 0.01, ^##^ *p* < 0.001 versus 3xTg + Vehicle group. All data passed the normality tests of Anderson‐Darling, D'Agostino‐Pearson, and Shapiro–Wilk tests. One‐way ANOVA with Tukey's test (A, B) and unpaired two‐tailed *t*‐test (D, E, G, I‐L) was used. PFC: prefrontal cortex; HP: hippocampus; EA: EAST36; MT: melatonin.

To further address the internal key driving the cerebrovascular protection of EAST36 in AD mice, this study explored MT regulation by EAST36 in AD intervention. Consistent with previous reports,^20^ our data showed MT moderately and significantly decreased in the PFC and HP regions of 3xTg‐AD mice (Figure 4A‐B). EAST36 remarkably improved the MT level of 3xTg‐AD mice. Nevertheless, the changes in MT level were not detected in the thalamus region (Figure S5), implying PFC and HP are more susceptible to MT deficit during AD development. Next, we examined whether the exogenous supplement of MT could mimic EAST36 effect on cerebrovascular repairment in 3xTg‐AD mice. The restoration of MT level after MT administration was verified in both PFC and HP regions of 3xTg‐AD mice (Figure 4D‐E). As expected, MT supplement rescued the hypoperfusion in 3xTg‐AD group (Figure 4F‐G). Structurally, MT supplement significantly suppressed the cerebral microvessels reduction in PFC and HP regions compared to 3xTg‐AD group (Figure 4H). It has been shown by the upregulation of vessel area fraction and vessel length density in the PFC region (Figure 4I‐J) and HP region (Figure 4K‐L). This protection by MT was comparable with EAST36 treatment in 3xTg‐AD mice, implying the importance of MT in cerebrovascular protection of EAST36.

### 3.5 Melatonin attenuated Aβ‐induced cerebrovascular endothelial cells impairment via melatonin receptor manner

**FIGURE 5:**
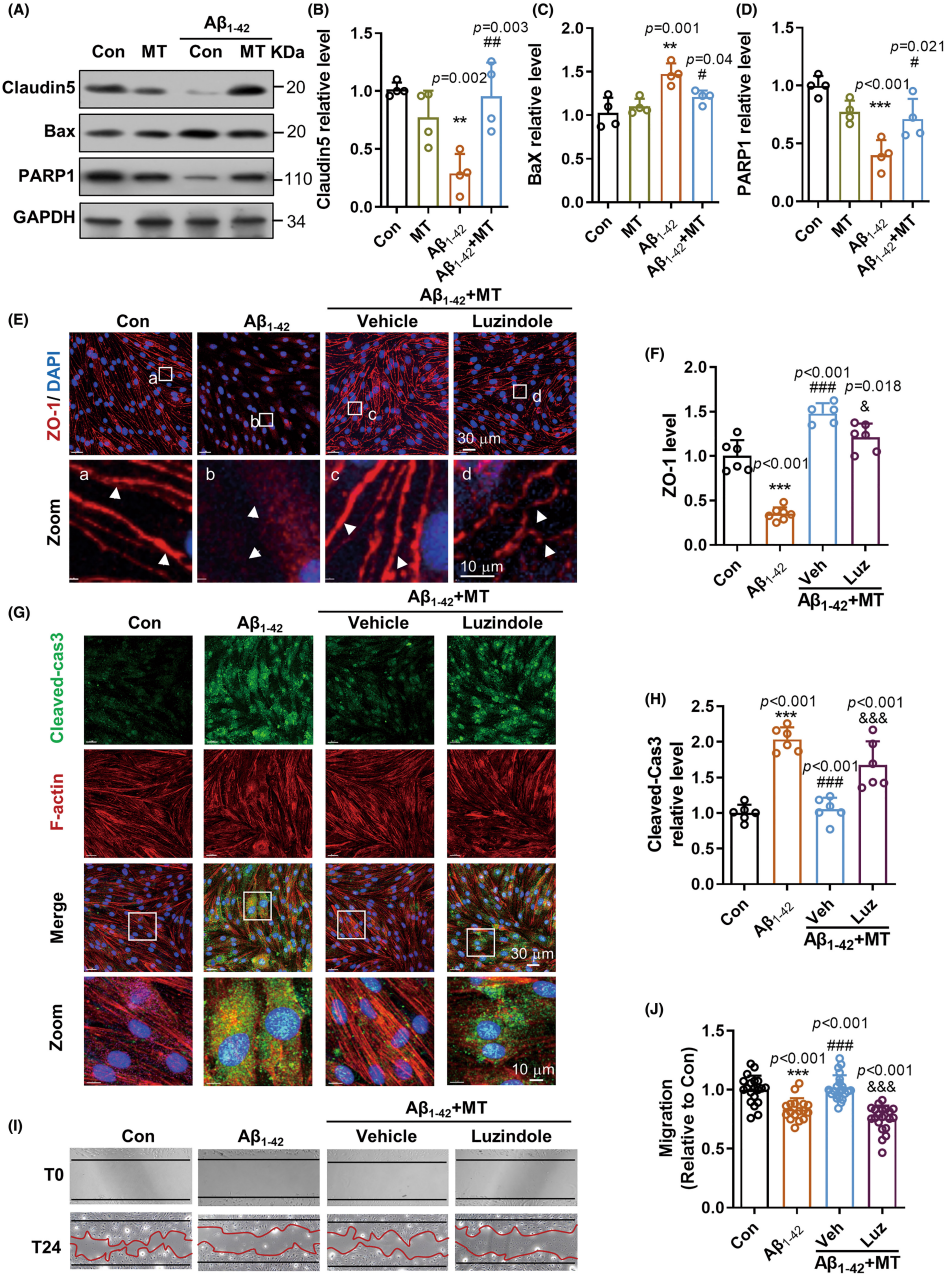
Melatonin attenuated Aβ‐induced cerebrovascular endothelial cells impairment via melatonin receptor manner. (A) Protein level changes of tight junction protein Cluadin5, apoptotic indicator Bax and PARP1 in mouse cerebrovascular endothelial cell bEnd.3 by Western blot analysis. GAPDH worked as the sample loading control. (B‐D) Quantification of protein level changes normalized to the Con group (*n* = 4). (E) Immunostaining of tight junction protein ZO‐1 (red) and DAPI (blue) in bEnd.3 cultures. The bottom panel is the zoomed views of white‐boxed regions. The breakdown of ZO‐1 around endothelial cells was indicated by the white arrow. (F) Fluorescence intensity of ZO‐1 was quantified and normalized to the Con group. (G) Immunostaining of cleaved‐caspase3 (green), F‐Actin (red), and DAPI (blue) in bEnd.3 cultures from each group. Arrow indicated the abnormal construction of F‐Actin in cells with increased cleaved‐caspase3. (H) Quantification of cleaved‐caspase3 fluorescence intensity in bEnd.3 cultures. *n* = 6. (I) Representative images of endothelial cell migration test before and after 24 h migration to assay the cell viability in each group. The boundary of migration before and after 24 h migration is indicated by the black and red lines, respectively. (J) Quantification of migration area in each group (*n* = 20 from 4 trials). Data were expressed as Mean ± SD. **p < 0.01, ***p < 0.001 versus Con group, ^#^ *p* < 0.05, ^##^ *p* < 0.01, ^###^ *p* < 0.001 versus Aβ_1‐42_ group. ^&^ *p* < 0.05, ^&&&^ *p* < 0.001 versus Aβ_1‐42_ + MT group. All data passed the normality tests of the Anderson‐Darling, D'Agostino‐Pearson, Shapiro–Wilk, and Kolmogorov–Smirnov tests. One‐way ANOVA with Tukey's test was used. Con: Control; MT: Melatonin; Luz: Luzindole; T0: Time at 0; T24: Time after 24 h.

Endothelial cell viability is required for cerebrovascular repair and angiogenesis. We next explored the mechanism of how cerebrovascular endothelial cells respond to MT by endothelial cell culture study in vitro. Consistent with findings in vivo, MT greatly prevented Aβ_1‐42_‐induced breakdown of tight junction components Claudin5 (Figure 5A‐B) and ZO‐1 (Figure 5 E‐F) in cerebrovascular endothelial cells. Moreover, MT significantly reversed the activation of these apoptotic molecules in cerebrovascular endothelial cells under Aβ_1‐42_ toxicity, as presented by the changes of apoptosis indicators Bax, PARP1, Caspase3 cleavage and F‐actin destruction (Figure 5A, C‐D, G‐H). Moreover, we observed the improvement of migration activity in endothelial cells responding to MT under Aβ_1‐42_ insult (Figure 5I‐J).

We further addressed whether MT receptor activation is involved in cerebrovascular endothelial cell protection. Immunoblotting showed bare changes in melatonin receptor 1 (MT1) and melatonin receptor 2 (MT2) protein levels in endothelial cells from each group (Figure S6). Notably, when suppressing MT1/ MT2 by antagonist Luzindole in endothelial culture, the protective effect of MT on cerebrovascular endothelial cells was impressively abolished (Figure 5 E‐J). Blockage of MT1/MT2 by Luzindole caused the elimination of tight junction protein ZO‐1 around cells (Figure 5 E‐F), enrichment of apoptosis marker cleaved‐caspase3 in cells (Figure 5G‐H), and inhibition of cell migration (Figure 5I‐J). Thus, these observations told that cerebrovascular protection of MT under AD pathology largely relied on the activity of melatonin receptors.

### 3.6 Blockage of melatonin receptors abolished the cerebrovascular protection of EAST36 in 3xTg‐AD mice

**FIGURE 6:**
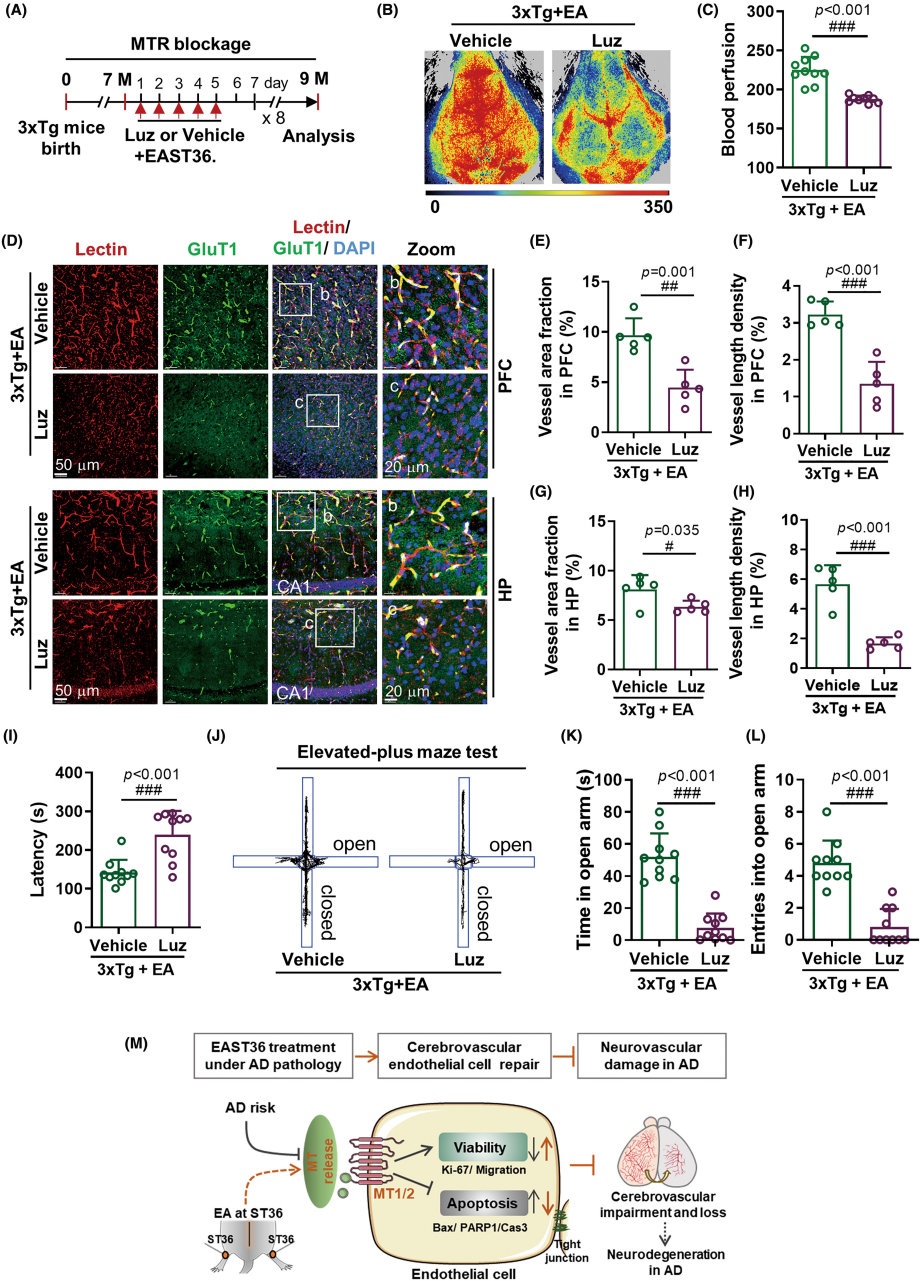
Blockage Of Melatonin Receptors Abolished The Cerebrovascular Protection Of East36 In 3xtg‐Ad Mice. (A) Schematic workflow of melatonin receptors blockage by Luzindole, and subsequent EAST6 treatment. (B, C) Representative images and quantification of cerebral perfusion in mice administered with vehicle or Luzindole 30 min before EAST36 (*n* = 10–12 mice). (D) Representative fluorescent images of Lectin (red) and GluT1 (green) and DAPI (blue) in PFC and HP of 3xTg mice treated with EAST36. (E‐H) Changes in cerebrovascular density in PFC and HP were presented as the vessel area fraction and vessel length density (*n* = 5 mice). (I) Changes of latency in food finding test (*n* = 10 mice). (J‐L) The representative trajectory of elevated plus maze test and the statistic (*n* = 10 mice). (M) Schematic diagram of the cerebrovascular protection of EAST36 in 3xTg AD mice. Data were expressed as Mean ± SD. ^#^ *p* < 0.05, ^##^ *p* < 0.01, ^###^ *p* < 0.001 versus Vehicle group. Data passed normality tests of Anderson‐Darling, D'Agostino‐Pearson, Shapiro–Wilk, and Kolmogorov–Smirnov test, except data in I and L. Unpaired two‐tailed *t*‐test with Welch's correction (C, H), unpaired two‐tailed *t*‐test (E, F, G, K) and nonparametric test with Mann Whitney test (I, L) were used. PFC: prefrontal cortex; HP: hippocampus; EA: EAST36; Luz: Luzindole; MTR: melatonin receptors.

Encouraged by the above findings, we next further clarified whether MT receptors were required for EAST36‐provoked cerebrovascular protection in vivo (Figure 6A). LSCI analysis revealed that Luzindole treatment greatly abolished the EAST36 effect on cerebral blood perfusion improvement in 3xTg‐AD mice (Figure 6B‐C). Moreover, cerebrovascular degeneration was also significantly exaggerated by Luzindole treatment compared with 3xTg‐AD mice treated with EA alone (Figure 6D), which was further quantified and presented as the reduction of vessel area fraction and vessel length density in the PFC region (Figure 6 E‐F) and HP region (Figure 6G‐H), respectively. Behaviorally, Luzindole significantly suppressed the EAST36‐improved olfactory function in 3xTg AD mice, as demonstrated by the increased food‐finding latency in food finding test (Figure 6I). Similar suppression was also found in the anxiety behavior assessment, as shown by the reduction in dwell time and visit times in open arms when compared with the vehicle group (Figure 6J‐L). Collectively, these findings addressed the robust protective effect of EAST36 is largely dependent on the endogenous MT/MT receptors signaling activation.

## 4 DISCUSSION

Effective intervention with low cost and side effects is urgently required to prevent AD onset and progression.^1^, ^2^ Accumulating evidence has addressed the impressive effect of acupuncture on mitigating cognitive deficits in AD.^14^, ^18^, ^19^, ^39^, ^40^ Here, this study supported it with new findings that EAST36 worked efficiently to promote cerebrovascular repair in both structure and function, thereby attenuating neurological disorders in 3xTg‐AD mice. In mechanism, the cerebrovascular protection of EAST36 majorly relied on the activation of MT signaling to improve cerebrovascular endothelial cell viability under AD pathology. Thus, our study suggested EAST36 as a robust and promising avenue for AD prevention and treatment (Figure 6M).

As one of the most commonly used acupoints to boost life energy flow and circulation, according to acupuncture theory, ST36 acupoints are endowed with a crucial role in disease treatment by modulating inflammation, oxidative stress, circadian rhythms, and immune system.^13^, ^19^, ^41^ Here, we clarified that ST36 stimulation could boost cerebrovascular recovery in AD conditions. Moreover, we found the stimulation frequency‐dependent protection of EAST36 against cerebrovascular injury. EAST36 with high frequency (dilatational stimulation, 20 Hz /100 Hz) significantly reboot the cerebrovascular function and thereby reversed the neurological symptom in the early stage of AD. In contrast, EAST36 with low frequency (dilatational stimulation, 2 Hz /10 Hz) seemed weak in the protective effect. This finding was also supported by previous reports on the stimulation frequency and effect. For example, EA at 100 Hz, not 2 Hz, efficiently increased dopaminergic neuron re‐generation in Parkinson's disease treatment.^42^ EA with 50 Hz worked much better in cognitive improvement in AD when compared with 30 Hz and 2 Hz.^14^ To our knowledge, the mechanisms might be associated with the frequency‐dependent driving of distinct neurotransmission and endogenous factors release.^14^, ^15^, ^42^, ^43^ Inspired by these findings, we speculate that the effective stimulation frequency of EA treatment varies depending on the somatic acupoints and disease state. Accordingly, the findings that EAST36 with high frequency has the advantage over low frequency suggested the merits of EA, in comparison with manual acupuncture, in precisely controlling the stimulation intensity and frequency.

Emerging evidence has highlighted the role of cerebrovascular dysfunction in the early stage of AD, leading to low blood perfusion, abnormal reduction of Aβ clearance, and neuronal dysfunction.^7^, ^38^, ^44^, ^45^ Cerebrovascular protection has attracted increasing attention as the target for AD treatment.^46^ Coincidently, we found that EAST36 significantly boosted cerebral perfusion in AD mice, in line with the improvement of neurological performance and cerebrovascular Aβ clearance in AD mice after EAST36. As we know so far, cerebral hypoperfusion is associated with multi factors in AD progression, including vascular contraction by smooth muscle cells and pericytes, blood–brain barrier breakdown, and capillary degeneration.^34^, ^47^ EA treatment could improve blood flow by enhancing NO‐releasing against stroke.^48^, ^49^ Therefore, besides our proposal that EAST36 promoted cerebrovascular endothelial cell repair in AD conditions, alternative factors, such as pericyte response and neuronal stimulation, are potentially involved in the EAST36 effect on cerebral perfusion in AD.

In addition, we found that compared with the dramatic enhancement of cerebrovascular density indicated by lectin, the recovery of cerebral perfusion after EAST36 was relatively moderate in the present study. It might be explained by the development manner of cerebrovascular structure and function in angiogenesis.^37^, ^50^ Thus, our results further suggested more vascular network is to be mature and engaged in blood circulation after 8‐weeks EAST36 treatment. Considering that olfactory dysfunction and neuropsychiatric symptoms mutually interact with AD progression and precede the vast neurodegeneration in the brain,^27^, ^51^, ^52^, ^53^ treatment of olfactory dysfunction and neuropsychiatric symptoms is not only the issue of life quality improvement for the patient but also the intervention of AD progression. Excitedly, our data suggested that EAST36 treatment worked in this position by relieving early neurological symptoms and Aβ accumulation in cerebral vessels in AD mice, greatly downregulating the risks of progressive cognitive decline in AD.^51^, ^52^, ^54^ Thus, the post‐effect of EAST36 is speculated to be positive based on the removal of risk factors and maturation of the newly formed vascular network.

MT protection in neurovascular dysfunction has long been recognized.^20^, ^21^ Large amounts of studies emphasized the importance and necessity of improving MT level and biofunction against aging, insomnia, anxiety, and neurodegenerative disease AD.^20^, ^55^, ^56^, ^57^ In this study, we clarified EAST36 as a modality to enhance MT signaling and successively stabilize the cerebrovascular network in AD mice. This action of EAST36 on MT was also supported by previous studies from insomnia treatment.^55^, ^56^ As we know, MT performs bio‐functions in receptor‐dependent or independent manner.^20^, ^21^, ^58^, ^59^, ^60^ Our study revealed the effect of EAST36 dramatically relied on MT receptors activation, as supported by the consequence of MT1/MT2 blockage. Furthermore, EAST36 partially restored the decreased MT1/MT2 level in PFC from AD mice (Figure S7). While EAST36‐initiated MT signaling activation on other neurovascular components, including neuronal cells and microglial cells, still could not be excluded in this study. As shown, MT receptor expression was observed in the neuronal cell, microglial cell, and endothelial cell (Figure S7), and the protection of MT receptor activation was also reported in neuronal cells.^61^, ^62^ These pieces of evidence suggest activation of MT signaling, involving MT release and activation of MT receptor, is required for the beneficial effect of EAST36 in neurovascular protection during AD pathology. While more exploration combined with cell type‐specific conditional knockout of MT receptors would be required to fully outline the signal transduction mechanism of EAST36 action on neurovascular protection against AD.

Subsequently, these observations raise the question of how acupuncture improves MT levels in the brain. Current evidence implicates MT could be synthesized and released from the pineal gland and extra‐pineal tissue including the gastrointestinal tract, retina, and skin under the driving by the noradrenergic projection of sympathetic neurons.^20^ Once released, MT surpasses the vessel into circulation and reaches target cells and tissue. Emerging research suggested MT synthesis also happens in subcellular organs and acts locally in mitochondria.^63^ To date, there is a sparking investigation of the pathway mediating EAST36‐evoked MT release. Our very preliminary data implicated EAST36 could enhance the MT synthesis enzyme AANAT expression in the intestine of AD mice and upregulated MT synthesis from 5‐HT in intestinal tissue (data not shown). More study is required to get conclusive evidence on the direct source of the EAST36‐enhanced MT release. This is the area we are pursuing in the following project.

## 5 CONCLUSION

In summary, our study illustrates the cerebrovascular protection of EAST36 in AD, providing credible evidence to support EAST36 as an effective approach for AD prevention and treatment. The findings of the frequency‐dependent effect of EAST36 would help improve clinical acupuncture practice in AD treatment. Moreover, our exploration of MT release and MT receptor signaling shed light on the mechanism deployed in EAST36‐evoked cerebrovascular recovery in AD progression.

## AUTHOR CONTRIBUTIONS

YM Jiang, YH Tan, and YS Lin conducted mice experiments and data acquisition. XK Shen conducted behavioral tests. MH Liao contributed to cell culture and data collection. H Wang and NN Lu contributed to data analysis and manuscript revision. F Han and NG Xu contributed to the project concept and design, manuscript revision, and funding support. CZ Tang, JX Song, and RR Tao contributed to the project concept and design, data interpretation, manuscript writing, and financial support. All authors have read and approved the final manuscript.

## CONFLICT OF INTEREST

All authors have declared no competing interests.

## Supporting information


AppendixS1

AppendixS2

## Data Availability

All data included in this study are available upon reasonable request by contacting the corresponding author.
