# Supplementary material for: Electroacupuncture ameliorates cerebrovascular impairment in Alzheimer's disease mice via melatonin signaling
Source: CNS Neurosci Ther. 2022 Nov 15;29(3):917–31. doi: 10.1111/cns.14027 (PMC9928543; doi:10.1111/cns.14027)
Supplement: Supplementary file 1 — AppendixS1 [file CNS-29-917-s002.docx]

**Supplemental information**

1. **Materials and methods**
   1. **Behavioral tests;**
   2. **Cerebral blood perfusion analysis;**
   3. **MT analysis by ELISA;**
   4. **Cell culture and treatment;**
   5. **Migration test;**
   6. **Fluorescent immunostaining;**
   7. **Western blot analysis;**
2. **Supplemental Figures**

**Figure S1.** Temporal changes of neurological performance in 3xTg-AD mice.

**Figure S2.** Effect of EAST36 at low frequency (2/10Hz) on neurological function and cerebral vascular perfusion in 3xTg-AD mice.

**Figure S3.** EAST36 suppressed Aβ_1-42_ deposits in 3xTg-AD mice rather than APP expression.

**Figure S4.** EAST36 suppressed neurovascular damage in 3xTg-AD mice.

**Figure S5.** Melatonin level in the thalamus region of mice brain tissue.

**Figure S6.** Melatonin receptor level changes in cerebral microvascular endothelial cells.

**Figure S7.** Melatonin receptors expression profile in the mouse brain.

**Figure S8.** Suppression of microglial activation in 3xTg-AD mice after EA treatment or MT treatment.

1. **Materials and methods**
   1. **Behavioral tests**

Food-finding test is the method for detecting olfactory symptoms in rodents as described previously. Briefly, mice were habituated in the test cage 24 h before the test and then were treated with food deprivation for 15 h before the test to improve motivation in food finding. Then, the food pellet (weight 1 g) was buried in a clean cage (50 cm x 40 cm x 40 cm) filled with new bedding (bedding depth 3 cm, while the pellet was placed 1 cm under the surface). The experimental mouse was placed at the center of the cage, then explored in the cage for 5 minutes. The latency to find the buried food pellet was recorded. If the mouse failed to find the food within 5 minutes, the latency was recorded as a maximum of 5 minutes.

Elevated-plus maze (EPM) was applied to assess anxiety in mice as described previously. Briefly, the elevated plus maze consisted of a plus-shaped maze with two open arms, two closed arms, and a center area and was placed 100 cm above the floor. Mice were placed in the center of the maze and freely explored the maze for 5 minutes. The trajectory of mice in the maze was recorded and the number of entries into open arms was calculated with the tracking system (Digbehv, Shanghai Jiliang Software Technology China). All mice were tested for one trial.

Morris-water maze test was employed to evaluate the memory and cognitive deficit in experimental mice. Briefly, a standard water maze with a 2 m diameter and 50 cm height was prepared, filled with water that powered non-fat milk. The tank was divided into four quadrants and the platform with a 10 cm diameter was positioned in the center of one target quadrant, hidden 2 cm below the water surface. All tests were performed at the same time each day in a dimly lit room with visual cues for each quadrant and recorded by the tracking system (Digbehv, Shanghai Jiliang Software Technology, China). The assessment consisted of 6-day trials including 5-day training with a hidden platform and 1-day probe test without a platform in the water. In the phase of training, the mouse performed 4 trials each day at 30-min intervals for 5 consecutive days. They were placed into the water from a random start quadrant and allowed to swim freely until they reached the hidden platform within 60 seconds. If failed, they were gently guided to the platform. The latency to reach the platform was recorded for learning curve analysis. In the probe test, the hidden platform was removed from the previous location. The mouse was placed into the water for one trial test from the direction farthest from the platform quadrant used in training days. The swimming trajectory within 60 seconds was recorded to analyze the platform cross times and time spent in the target quadrant. At the end of each trial, the mice were removed, dried, and returned to homecages.

**1.2 Cerebral blood perfusion analysis**

Laser speckle contrast imaging (LSCI) was applied to measure the cerebral blood flow with PeriCam PSI System (Perimed Inc, US) following the manufacturer’s instructions. Briefly, mice were anesthetized by isoflurane and then transferred to the stereotaxic apparatus equipped with a heat blanket at 37 °C. Shaved fur and made an incision along the midline to expose the skull. The laser speckle imaging was set up at a display rate of 2 images/s for 30-second blood flow recording including 10-second baseline imaging. The skin covering the exposed skull was stitched up after imaging and mice were carried back to homecages. The blood perfusion images were presented as heat maps with a scale ranging from 0 to 350 and the blood flow dynamics were analyzed with the software PIMSoft (Perimed Inc, US).

**1.3 MT analysis by ELISA**

The mouse brain tissues from the hippocampus and prefrontal cortex were dissected and stored in dry ice immediately from 9:00 am-11:00 am, which was followed by ELISA detection according to the provided manual of the MT ELISA kit (E4630, BioVision, US). Briefly, tissue samples were homogenized in lysis buffer and centrifuged at 140000 r/min for 30 min at 4 ℃. Then, the lysate was collected for MT analysis. The data was presented as MT level/brain tissue weight (pg/10 mg).

**1.4 Cell culture and treatment**

Mouse brain microvascular endothelial cells (bEnd.3, ATCC, US) were cultured as previously. Briefly, cells were cultured in Dulbecco's Modified Eagle's Medium (DMEM) (11995065, Thermo Fisher, US) with 10% Fetal bovine serum (FBS) (0500, ScienCell, US) and then incubated in cell culture incubator with 5% CO2 at 37 ℃. For the Western blot analysis, cells were cultured in culture flasks and treated with 10 μM Aβ_1-42_ oligomer (RP10017, Genscript, China), 10 μM MT (M5250, Sigma Aldrich, US), or solvent vehicle (0.1% DMSO in PBS) for 48 hours. In sample preparation for fluorescent immunostaining, cells were plated on Poly-L-Lysine (0403, ScienCell, US)-coated glass slice overnight and then treated with Aβ_1-42_ oligomer, MT for 48 hours. In the migration test, cells were cultured in a 6-well culture plate and subjected to subsequent treatments. For Luzindole treatment, Luzindole (10 μM, L0316, TCI America, US) was added to cells 30 mins before MT treatment. Cells treated with PBS work as the vehicle group.

**1.5 Migration test**

The migration activity of bEnd.3 endothelial cells from each experimental group were evaluated by wounding analysis. Briefly, when cell cultures reach 100% confluence in the culture plate, the cell wound was made by scraping cells off with a scraper. After that, cell cultures were washed to remove the scraped cells and the wound in each group was captured as baseline T0. Then cell cultures were incubated at 37 ℃ in culture medium without FBS and with the addition of Aβ_1-42_ oligomer, MT, or Luzindole as described above. After 24 hours of migration, cells were fixed with 4% PFA, and wound T24 was imaged for quantification. The migration activity of the cells was quantified as the change ratio of wound area ((T0-T24) /T24) in each field, and then the data were normalized to the control group that was treated with PBS.

**1.6 Fluorescent immunostaining of brain tissues and cell culture sample**

As previously described, experimental mice were anesthetized and perfused with PBS and then 4% paraformaldehyde solution (PFA). The whole brain was isolated from the skull and fixed in 4% PFA at 4 ℃ overnight. After that, the brain was immersed in 30% sucrose solution at 4 ℃ for two days. Frozen brain blocks were cut into 40 μm-thick sections with cryostat sectioning. Brain sections were washed with PBS 3 times and permeabilized with 0.1% Triton X-100 for 30 min, and then incubated in blocking buffer for 1 hour at room temperature. After blocking, brain slices were incubated with primary antibody Aβ_1-42_ (NBP2-13075, Novus Biologicals, US), NeuN (ab104224, Abcam, England), GluT1 (12939, Cell signal technology, US), Ki67 (PA5-19462, Invitrogen, US), ZO-1 (ab96587, Abcam, England), cleaved-caspase3 (9661, Cell signal technology, US) or DyLight594 Tomato Lectin (DL-1177, Vector Labs, US), DyLight554 F-actin (13054, Cell signal technology, US) at 4 ℃ overnights. After washing 3 times, sections were incubated with Alexa fluor-secondary antibodies (Invitrogen, US) for 4 hours at room temperature followed by 10 minutes of staining with DAPI. For Nissl staining, brain sections were incubated with NeuroTrace-fluorescent Nissl stains (MP21480, Thermo Fisher, US) for 20 minutes following NeuN staining. Finally, slices were mounted with the antifade medium containing DAPI (H1200, Vector Labs, US). For cell fluorescent staining, cell samples were fixed with 4% PFA after culture and collection. Then they were conducted with fluorescent immunostaining following the above steps. The fluorescence images and z-stack images were acquired by Nikon A1R confocal laser scanning microscope. Imaris and Image J were used for 3D-reconstructed images and image analysis.

**1.7 Western blotting analysis of protein level**

The brain tissue from deeply anesthetized mice was quickly removed and dissected to get hippocampus and prefrontal cortex tissue samples. For cell culture samples, the cells after treatment were collected by scraping them off from culture flasks. Then, samples were collected and applied for western blot analysis. Then, the protein samples were extracted and prepared on ice with lysis buffer containing cocktail protease inhibitors (P8340, Sigma-Aldrich, US). The protein lysates were collected and estimated for protein concentration using Bradford's solution kit (P0006, Beyotime, China) after centrifuging at 140000 r/min for 30 min at 4 ℃. In Western blotting analysis, equal amounts of protein were loaded and separated by sodium dodecyl sulfate-polyacrylamide (SDS-PAGE) gel electrophoresis and then transferred to PVDF membrane for 1 h at 200 mA. Membranes were blocked in 5% fat-free milk in Tris‐buffered saline containing 0.05% Tween‐20 (TBST). Proteins were detected by specific primary antibodies against Bax (2772, Cell Signal Technology, US), PARP1 (9532, Cell Signal Technology, US), Claudin5 (35-2500, Invitrogen, US), cleaved-caspase3 (9661, Cell signal technology, US), and GAPDH (70-Mab5465-040, Multi-Sciences Biotech, China). All blots were incubated with the primary antibodies in blocking buffer overnight at 4°C. After incubation and three-time washes in TBST, membranes were incubated with the following HRP-linked secondary antibodies (7074S, 7076S, Cell Signal Technology, US). The bands were visualized by enhanced chemiluminescence (34580, Thermo Fisher Scientific, US) and quantified using Image J.

1. Su**pplementary figures**


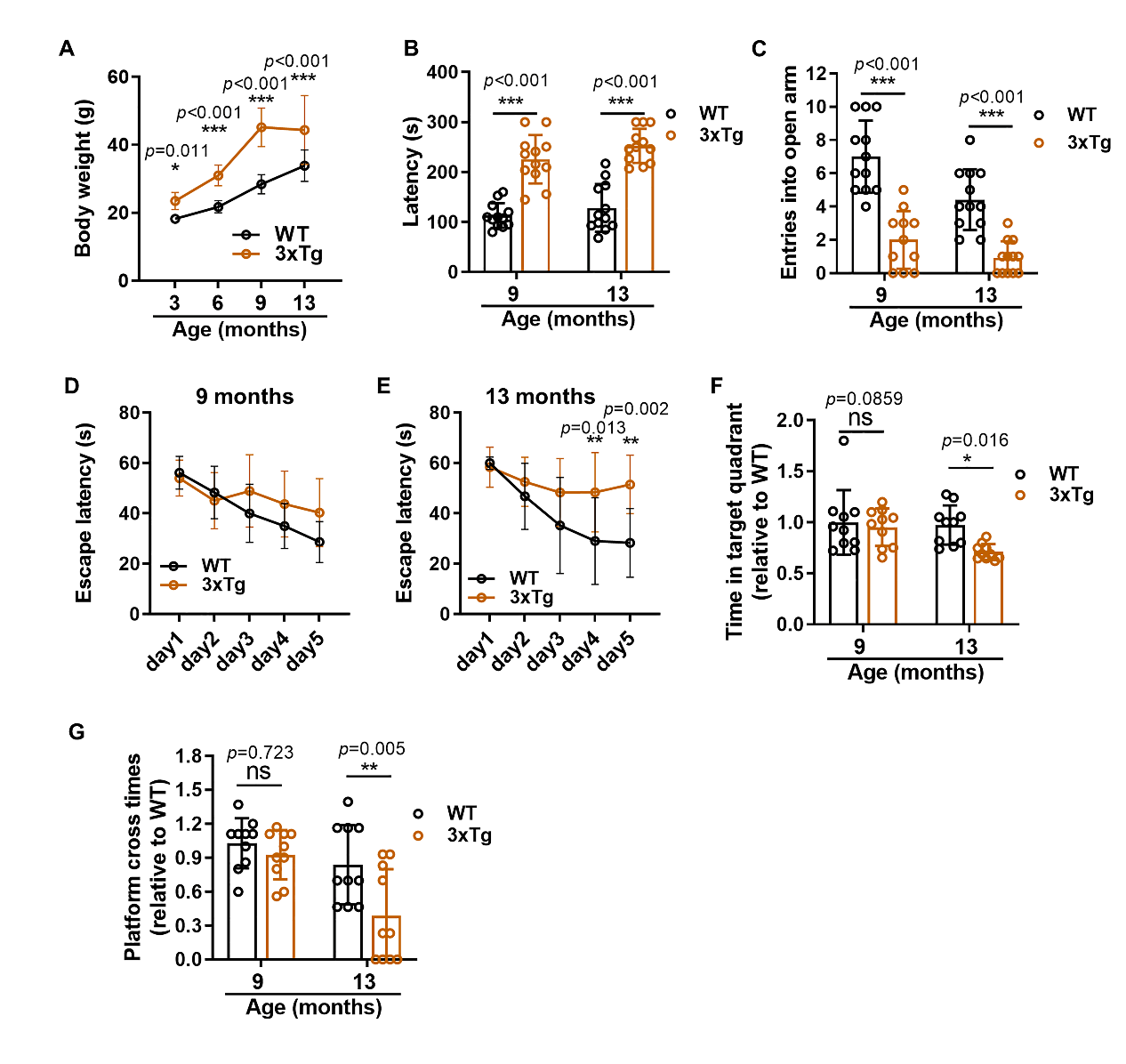


**Figure S1. Temporal changes of neurological performance in 3xTg-AD mice.** (A) Development of body weight in wild-type mice and 3xTg-AD mice from 3 months to 13 months. (B) The latency to find food in the food finding test. (C) Statics of entries into open arm in the elevated plus maze test. (D-G) Morris-water maze test was applied and presented as the learning curve during the training phase (D, E), time in the target quadrant (F), and times crossing platform location (G) during the probe phase. Data were expressed as Mean ± SD. *p < 0.05, **p < 0.01, ***p < 0.001 versus WT group. Data passed normality tests of Anderson-Darling (E-G), D’Agostino-Pearson (B-G), Shapiro-Wilk (A, E-G), and Kolmogorov-Smirnov test (F-G). Two-way ANOVA with the Sidak test was used. n = 10-12. ns: non-significant.

**
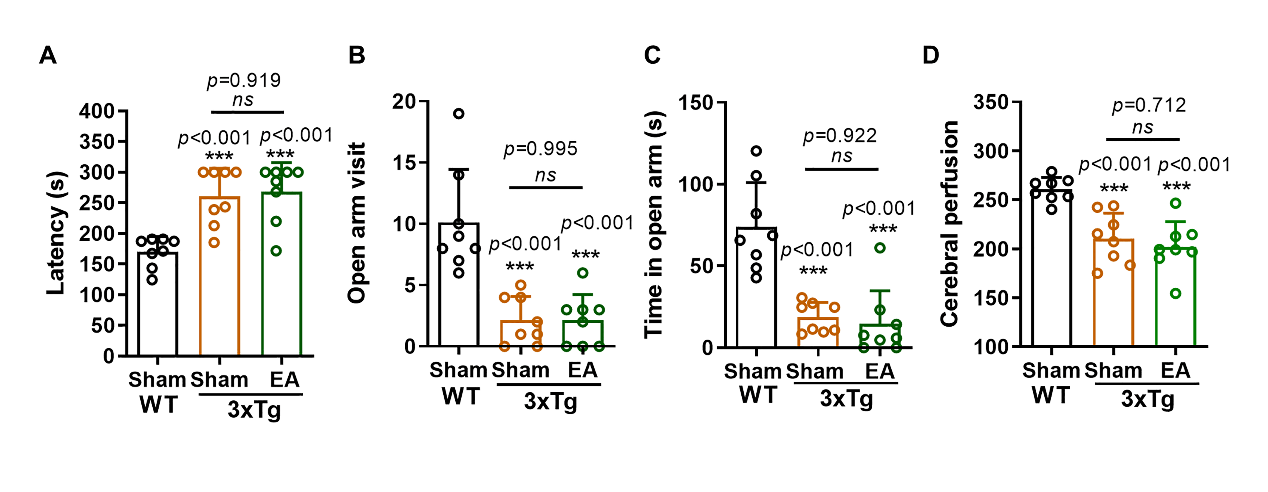
**

**Figure S2. Effect of EAST36 at low frequency (2/10Hz) on neurological function and cerebral vascular perfusion in 3xTg-AD mice.** (A) The latency to find food in the food finding test. (B, C) The statistic of the elevated plus maze test, as presented as the visit times in the open arm and time staying open arm. (D) The changes of cerebral perfusion in mice detected by LSCI. *n=8* mice. Data were expressed as Mean ± SD. ***p < 0.001 versus WT+Sham group. Data passed normality tests of Anderson-Darling (B, D), D’Agostino-Pearson (A, B, D), Shapiro-Wilk (B, D), and Kolmogorov-Smirnov test (C). A one-way ANOVA with Tukey’s test was used. n=10-12. ns: non-significant.


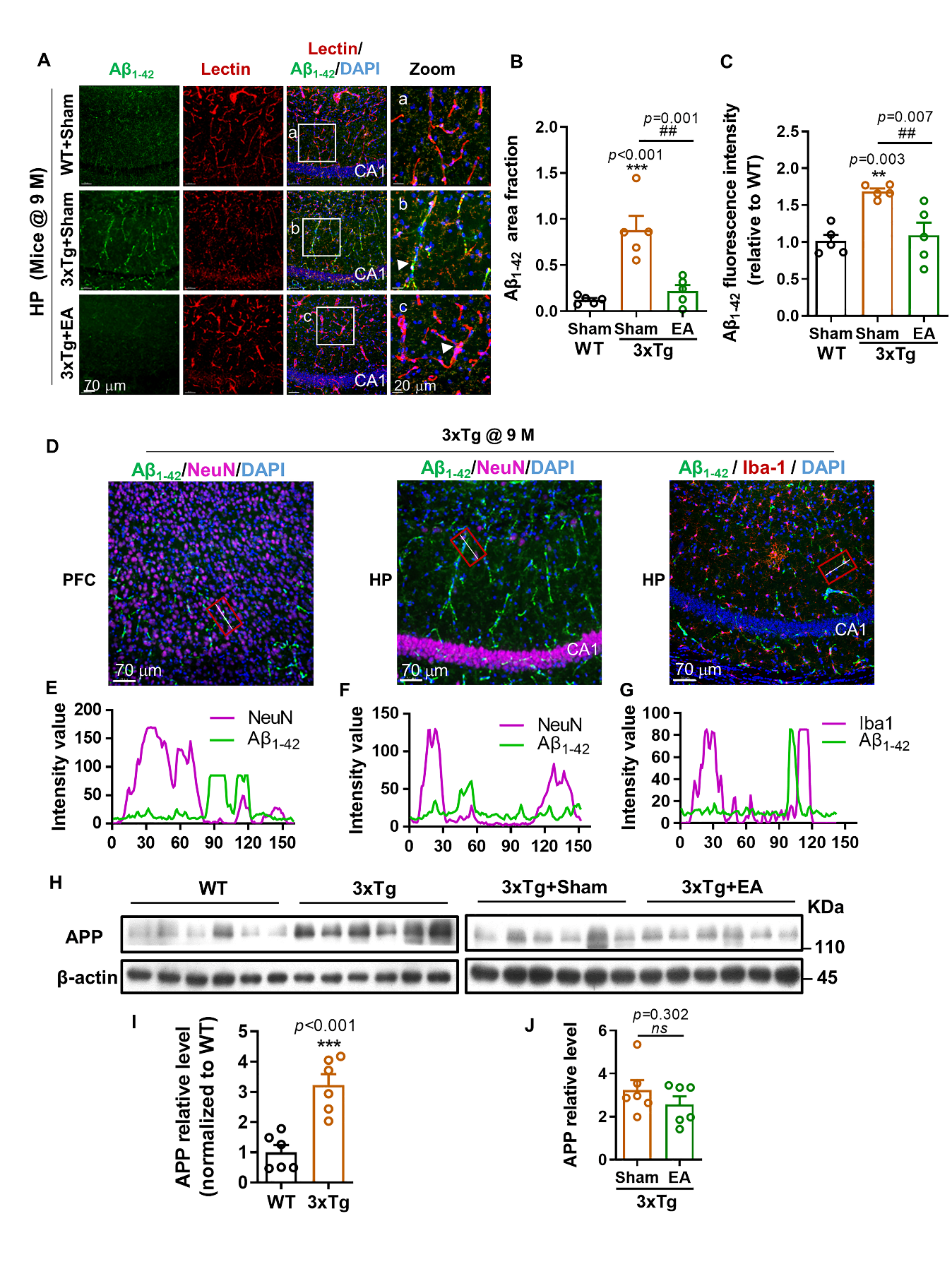


**Figure S3. EAST36 suppressed Aβ_1-42_ deposits in 3xTg-AD mice rather than APP expression.** (A) Distribution of Aβ deposits in HP region of mice. Aβ deposit was labeled with Aβ_1-42_ antibody (green). Cerebral microvessels were labeled with Lectin (red). DAPI (blue) was used for nuclei staining. White-boxed regions were enlarged in the right Zoom panel, respectively. (B, C) Quantitative analysis of Aβ deposit which was expressed as changes of Aβ deposit area fraction and fluorescence intensity (*n=*5 mice). (D) Representative images of Aβ_1-42_ (green) and neuron marker NeuN (rose red), or Aβ_1-42_ (green) and microglia marker Iba-1 immunostaining in PFC and HP from 3xTg-AD mice. (E-G) Distribution profile analysis of Aβ_1-42_ (green) and NeuN (rose red) or Iba-1 (red) along the white line in red boxes. (H-J) The protein level of APP full length in wild-type mice, 3xTg mice, and EA-treated 3xTg mice by Western blotting analysis (*n=*6 mice). Data were expressed as Mean ± SD. ***p* < 0.01, ****p* < 0.001 versus WT group, ^##^*p* < 0.01 versus 3xTg+Sham group. Data passed normality tests of Anderson-Darling (C, I-J), D’Agostino-Pearson (C, I-J), Shapiro-Wilk (B-C, I-J), and Kolmogorov-Smirnov test (B-C, I-J). One-way ANOVA with Tukey’s test (B, C) and unpaired two-tailed t-test (I, J) were used. APP: Aβ precursor protein; ns: non-significant.


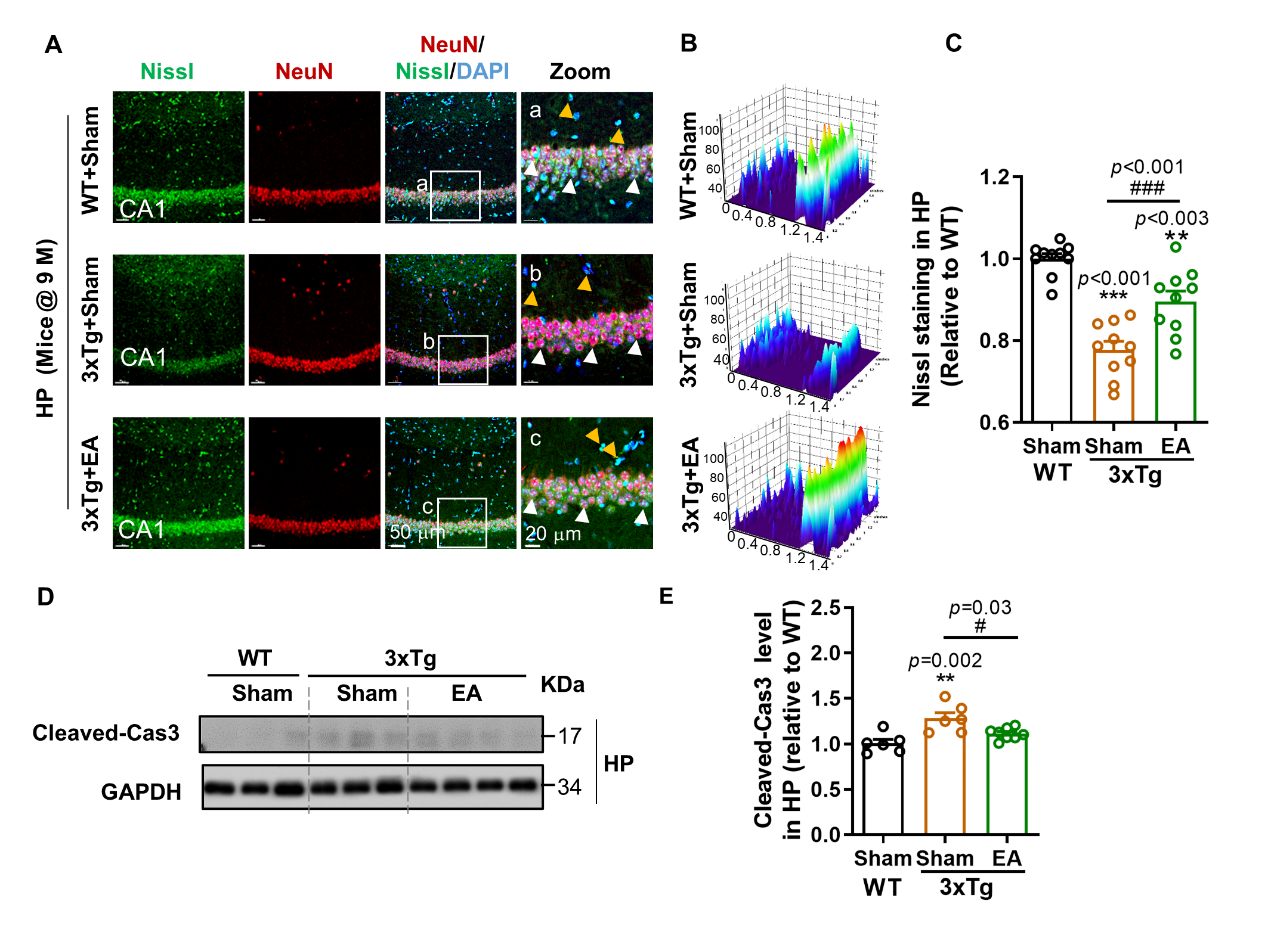


**Figure S4. EAST36 suppressed neurovascular damage in 3xTg-AD mice.** (A) Representative images of Nissl staining in the HP region of mice. The co-staining of Nissl (green), NeuN (red), and DAPI (blue) demonstrated Nissl level in neuron cells (white arrow). The co-staining of Nissl (green) and DAPI (blue) without NeuN signal showed Nissl levels in non-neuron cells (yellow arrow). (B) 3D surface plot analysis of Nissl fluorescence intensity changes in HP. (C) Nissl level was quantified and plotted as mean fluorescence intensity normalized to WT group (*n* =10 from 5 mice). (D) Western blotting analysis of cleaved-Caspase 3 in HP region. GAPDH worked as the sample loading control. (E) Protein level was quantified and normalized to WT group (*n* = 6-8 mice). Data were expressed as Mean ± SD. ***p* < 0.01, ****p* < 0.001 versus WT+ Sham group, ^#^*p* < 0.05, ^###^*p* < 0.001 versus 3xTg+ Sham group. Data passed the normality tests of Anderson-Darling, D’Agostino-Pearson, Shapiro-Wilk, and Kolmogorov-Smirnov tests. One-way ANOVA with Tukey’s test was used. HP: hippocampus; EA: EAST36.

**Figure S5. Melatonin level in the thalamus region of mice brain tissue.** The change in MT level was not observed among groups by melatonin Elisa analysis. Data were expressed as Mean ± SD. *n*=8. Data passed the normality tests of Anderson-Darling, D’Agostino-Pearson, Shapiro-Wilk, and Kolmogorov-Smirnov tests. One-way ANOVA with Tukey’s was used. EA: EAST36.


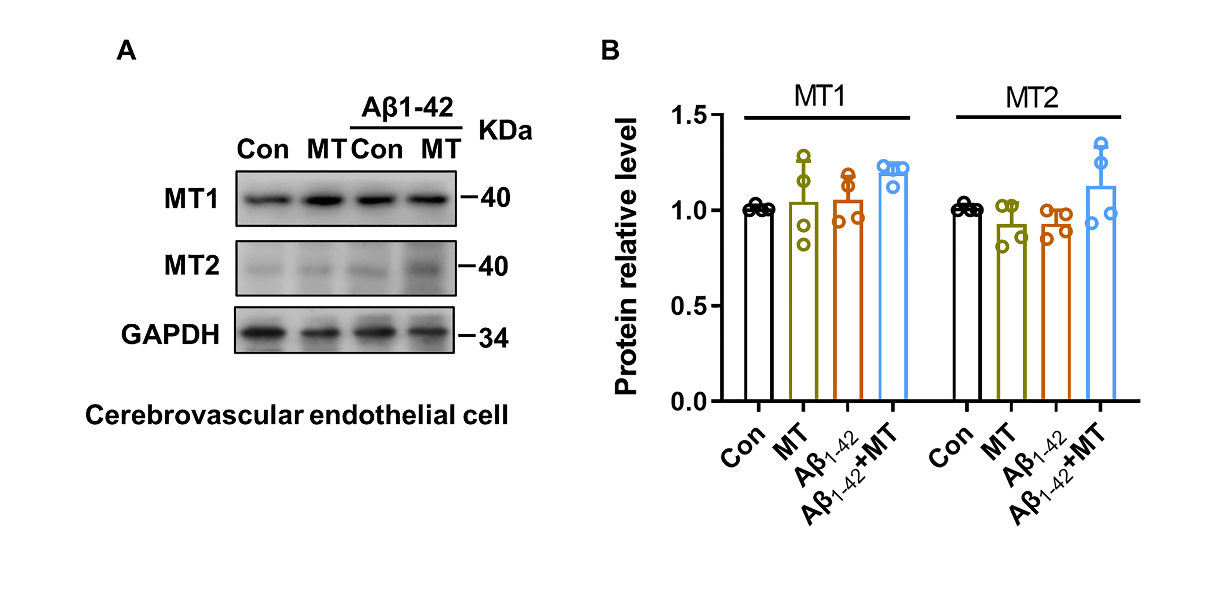


**Figure S6. Melatonin receptor level changes in cerebral microvascular endothelial cells.** (A) Western blot bands of MT1 and MT2 protein expression from bEnd.3 cultures after Aβ insult without and with Melatonin incubation. (B) Statistic of MT1 and MT2 protein level from Western blot bands. Data were expressed as Mean ± SD. *n*=4. Data passed the normality tests of Anderson-Darling, D’Agostino-Pearson, Shapiro-Wilk, and Kolmogorov-Smirnov tests. One-way ANOVA with Tukey’s test was used. MT: melatonin.


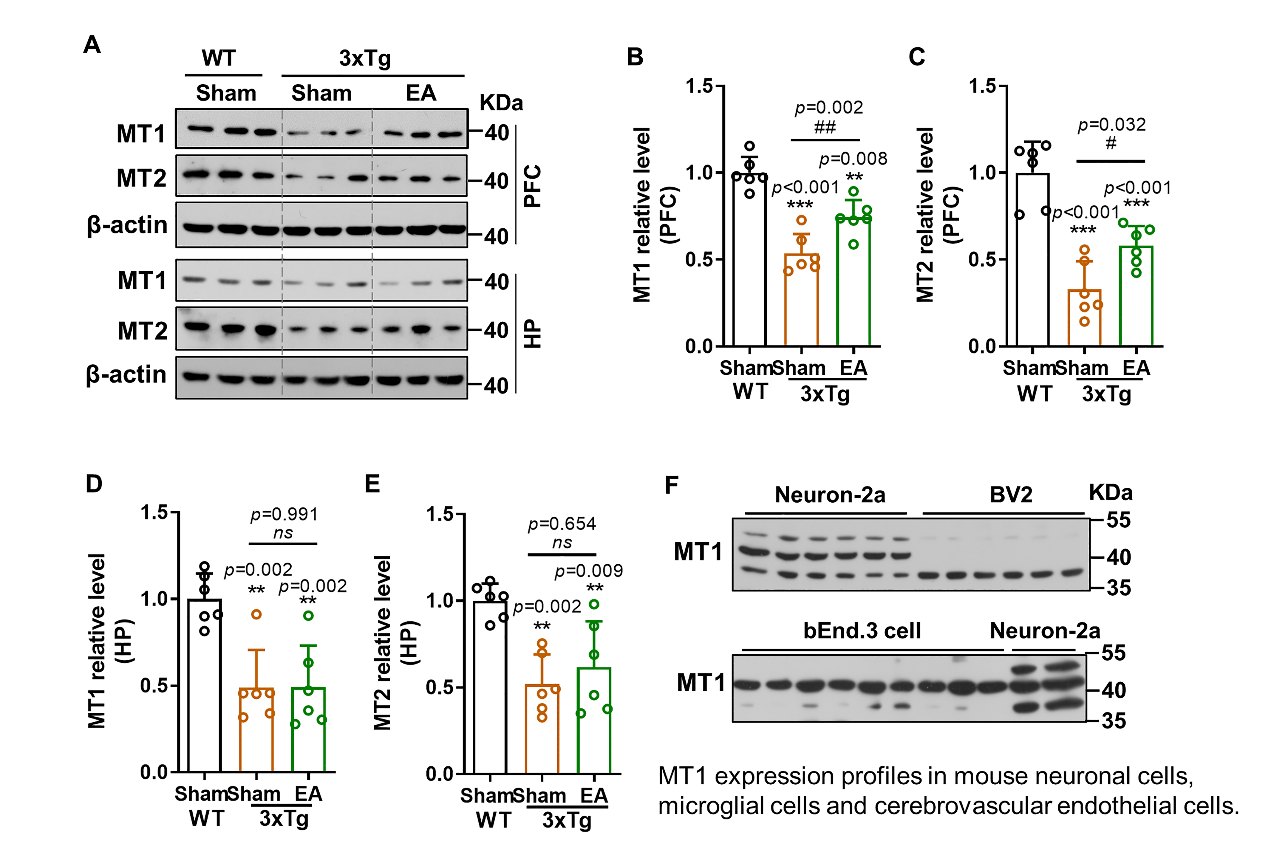


**Figure S7. Melatonin receptors expression profile in the mouse brain.** (A) Western blot bands of melatonin receptor 1 (MT1) and melatonin receptor 2 (MT2) from PFC and HP regions of wild-type mice and 3xTg AD mice without and with EA treatment at age of 9 months. (B-E) Statistics of the protein levels from Western blot bands in A. (F) Expression profile of MT1 in neuronal cells (Neuron-2a), microglial cells (BV2), and cerebrovascular endothelial cells (bEnd.3). Data were expressed as Mean ± SD. ***p* < 0.01, ****p* < 0.001 versus WT+sham group, ^#^*p* < 0.05, ^##^*p* < 0.01 versus 3xTg+ sham group. *n=*6 mice. Data passed normality tests of Anderson-Darling (B-C, E), D’Agostino-Pearson (B-E), Shapiro-Wilk (B-C, E), and Kolmogorov-Smirnov (B-C, E) test. Data in passed normality tests of Anderson-Darling, D’Agostino-Pearson, Shapiro-Wilk, and Kolmogorov-Smirnov test. The one-way ANOVA with Tukey’s test was used. PFC: prefrontal cortex; HP: hippocampus; EA: EAST36. ns: non-significant.


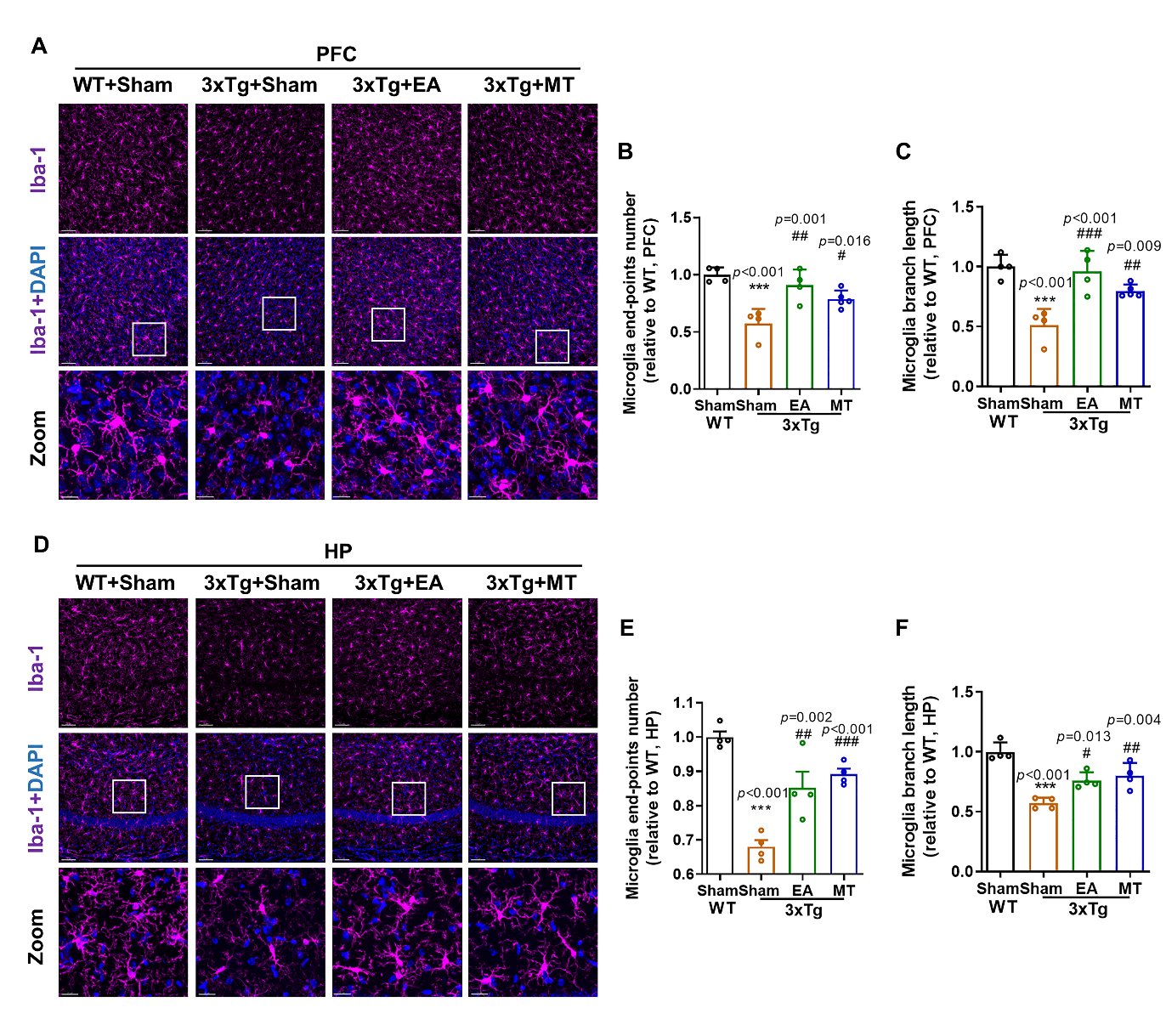


**Figure S8. Suppression of microglial activation in 3xTg-AD mice after EA treatment or MT treatment.** (A, D) Alternations of microglial morphology in the PFC region and HP region of mouse brains. (B, C, E, F) Statistic of the changes in microglia end-point number (B, E) and branch length (C, F). Data were expressed as Mean ± SD. *n*=4. Data passed the normality tests of Anderson-Darling, D’Agostino-Pearson, Shapiro-Wilk, and Kolmogorov-Smirnov tests. One-way ANOVA with Dunnett's multiple comparisons test was used. PFC: prefrontal cortex; HP: hippocampus; EA: EAST36; MT: melatonin.
