# Supplementary material for: Electroacupuncture ameliorates cerebrovascular impairment in Alzheimer's disease mice via melatonin signaling
Source: CNS Neurosci Ther. 2022 Nov 15;29(3):917–31. doi: 10.1111/cns.14027 (PMC9928543; doi:10.1111/cns.14027)

Full unedited blot for Figure 3G

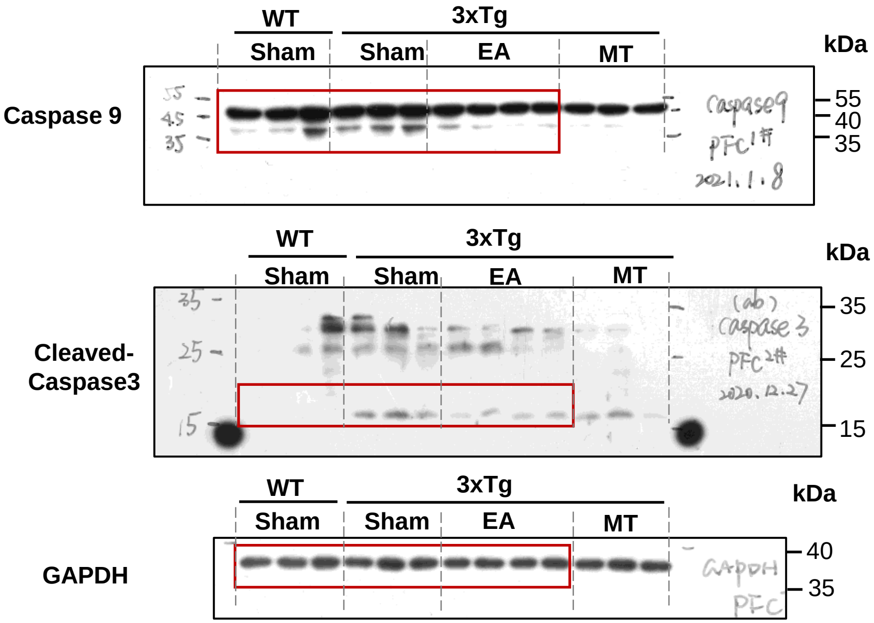

Full unedited blot for Figure S 3H

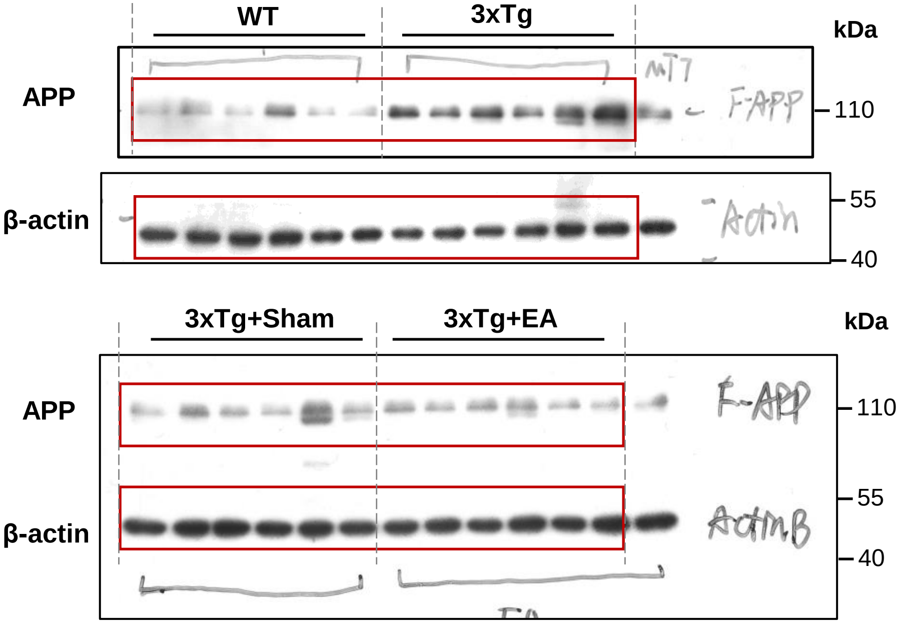

Full unedited blot for Figure S 4D

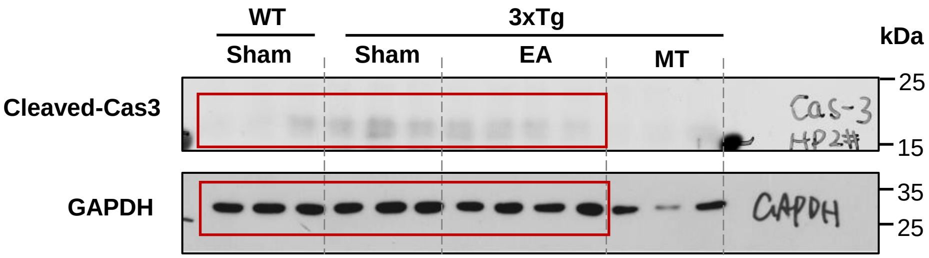

Full unedited blot for Figure 5A

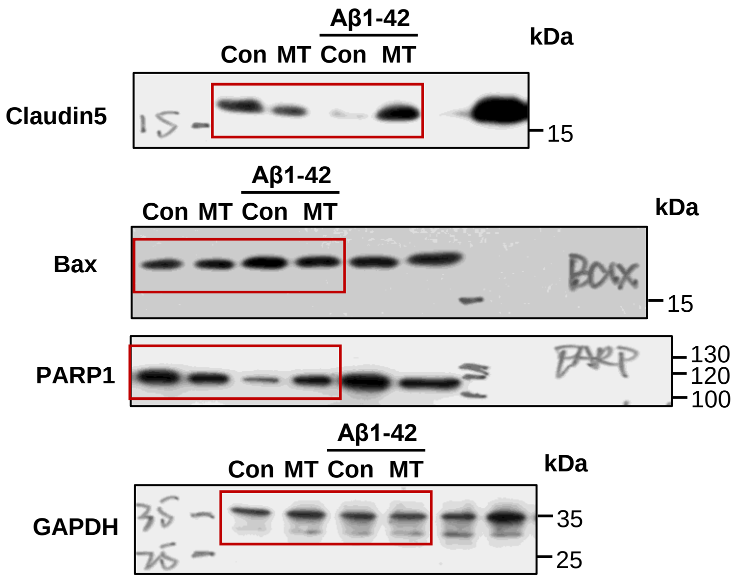

Full unedited blot for Figure S6A

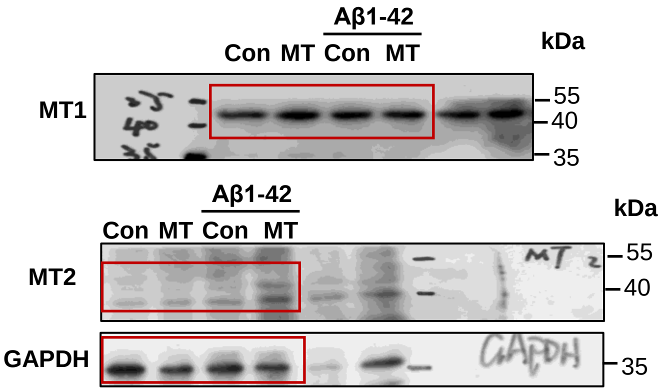

Full unedited blot for Figure S7A

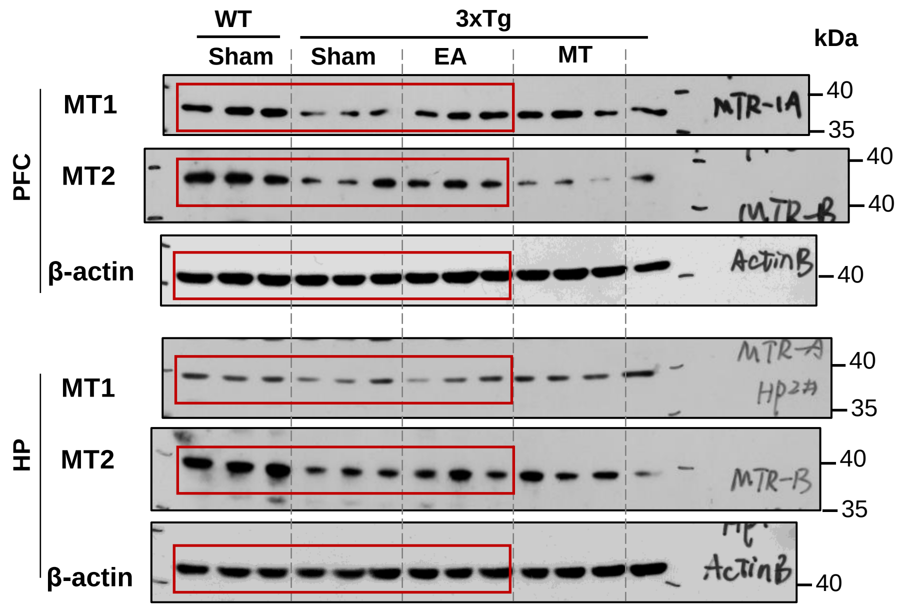

Supplement: Supplementary file 2 — AppendixS2 [file CNS-29-917-s001.pdf]
